# The Pentose Phosphate Pathway Regulates the Circadian Clock

**DOI:** 10.1016/j.cmet.2016.07.024

**Published:** 2016-09-13

**Authors:** Guillaume Rey, Utham K. Valekunja, Kevin A. Feeney, Lisa Wulund, Nikolay B. Milev, Alessandra Stangherlin, Laura Ansel-Bollepalli, Vidya Velagapudi, John S. O’Neill, Akhilesh B. Reddy

**Affiliations:** 1 University of Cambridge Metabolic Research Laboratories, Wellcome Trust-MRC Institute of Metabolic Science, Addenbrooke’s Hospital, Cambridge CB2 0QQ, UK; 2 Metabolomics Unit, Institute for Molecular Medicine Finland (FIMM), 00290 Helsinki, Finland

## Abstract

The circadian clock is a ubiquitous timekeeping system that organizes the behavior and physiology of organisms over the day and night. Current models rely on transcriptional networks that coordinate circadian gene expression of thousands of transcripts. However, recent studies have uncovered phylogenetically conserved redox rhythms that can occur independently of transcriptional cycles. Here we identify the pentose phosphate pathway (PPP), a critical source of the redox cofactor NADPH, as an important regulator of redox and transcriptional oscillations. Our results show that genetic and pharmacological inhibition of the PPP prolongs the period of circadian rhythms in human cells, mouse tissues, and fruit flies. These metabolic manipulations also cause a remodeling of circadian gene expression programs that involves the circadian transcription factors BMAL1 and CLOCK, and the redox-sensitive transcription factor NRF2. Thus, the PPP regulates circadian rhythms via NADPH metabolism, suggesting a pivotal role for NADPH availability in circadian timekeeping.

## Highlights

• Pentose phosphate pathway regulates circadian oscillations through NADPH metabolism
• Inhibition of pentose phosphate pathway remodels circadian gene expression
• NRF2 connects redox oscillations to transcriptional rhythms
• Pentose phosphate pathway modulation alters rhythmic behavior and tissue clocks

## Graphical Abstract

**Figure:**
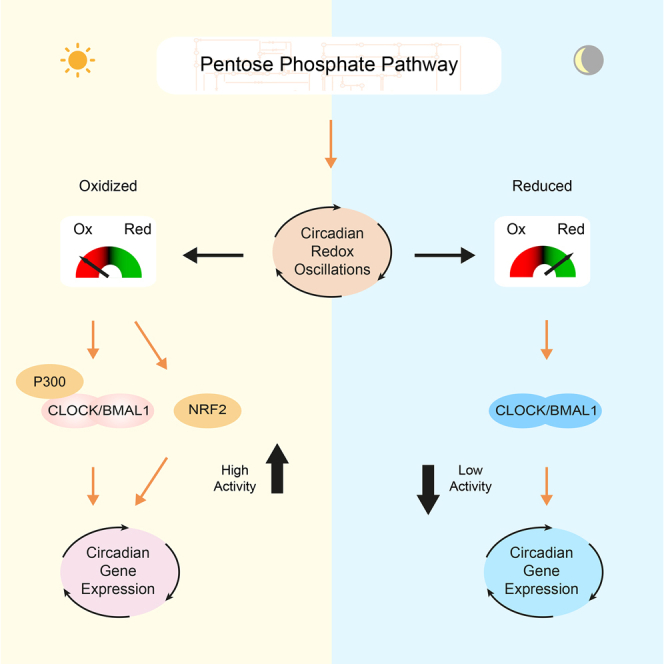

## Teaser

Current models of circadian clock control emphasize transcriptional networks. Here, Rey et al. identify the pentose phosphate pathway (PPP), which generates NADPH, as an important regulator of redox and transcriptional oscillations. Inhibition of this highly conserved metabolic pathway affects circadian rhythms in flies, mice, and human cells.

## Introduction

Mammalian models of the circadian clock center on transcription-translation feedback loop mechanisms, involving the core transcription factors BMAL1 and CLOCK (Bass, 2012). However, recent evidence has uncovered the existence of transcription-independent mechanisms of circadian timekeeping (Cho et al., 2014, Nakajima et al., 2005, O’Neill and Reddy, 2011, O’Neill et al., 2011). These likely preceded the existence of transcriptional oscillations during evolution, as highlighted by rhythms in the oxidation and reduction of peroxiredoxin proteins in a range of phylogenetically disparate organisms ranging from bacteria to humans (Edgar et al., 2012). In a simple model of non-transcriptional circadian oscillations, the red blood cell, oxidation cycles occur in association with robust circadian oscillations of the core cellular reductants NADH and NADPH (O’Neill and Reddy, 2011).

**Figure 1:**
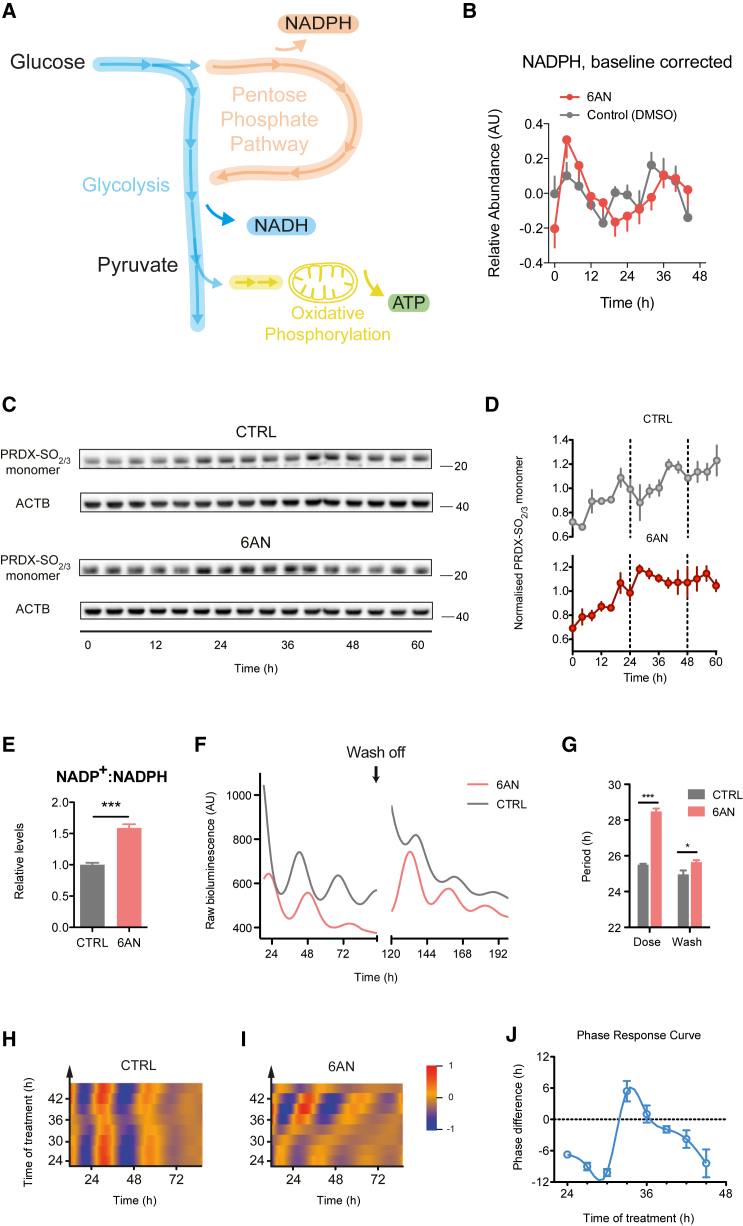
The PPP Regulates Redox and Transcriptional Oscillations in Human Cells (A) Schematic of glucose metabolism showing glycolysis, the pentose phosphate pathway (PPP), and oxidative phosphorylation in mitochondria. (B) NADPH levels in *Bmal1*:*luc* U2OS cells treated with 5 mM 6-aminonicotinamide (6AN) versus control (DMSO) for 2 consecutive days (mean ± SEM, n = 3–4). (C) Representative immunoblots showing overoxidized peroxiredoxin (PRDX-SO_2/3_) monomers with loading controls (β-actin, ACTB) for *Bmal1*:*luc* U2OS cells treated with 5 mM 6AN versus control (DMSO). Molecular weights (kDa) shown on right side of blots. (D) Quantification by densitometry of immunoblots from (C). Values were normalized to the average for each blot (mean ± SEM, n = 3). (E) NADP^+^:NADPH ratio of cells treated with 6AN (mean ± SEM, n = 3–4; two-tailed Student’s t test; ^∗∗∗^p < 0.001). (F) Bioluminescence traces for *Bmal1*:*luc* U2OS cells treated with 5 mM 6AN versus control (DMSO), followed by wash off after 96 hr. (mean values shown, n = 3–6). (G) Quantifications of the period length from (F) before and after wash off (mean ± SEM, n = 3–6; two-tailed Student’s t test; ^∗∗∗^p < 0.001, ^∗^p < 0.05). (H and I) Heatmaps showing bioluminescence traces for *Per2*:*luc* U2OS cells treated at the indicated time points with 5 mM 6AN (I) or control (DMSO) (H) until the end of the experiment. Each row represents a different time of treatment. (J) Phase-response curve showing the phase shifts caused by treatment with 6AN compared to control (DMSO) at different time of the day (mean ± SEM, n = 3–6).

In central carbon metabolism, glycolysis and the pentose phosphate pathway (PPP) oxidize glucose to produce NADH and NADPH, respectively (Figure 1A). These pathways are common to most aerobic organisms and produce an important fraction of the cellular pool of NAD(P)H (Fan et al., 2014). Since the peroxiredoxin oxidation cycle is directly influenced by the availability of NADPH (Wood et al., 2003), we hypothesized that these cellular reduction pathways might regulate redox and transcriptional oscillations in nucleated cells.

Using a combination of pharmacologic and genetic approaches, we found that inhibition of the PPP altered circadian rhythms in human cells. We observed similar effects in mouse tissues, and we also found that PPP inhibition affected the pattern of rhythmic behavior in *Drosophila*. Our study indicates that the interplay between redox and transcriptional cycles relies on the circadian transcription factors BMAL1/CLOCK and the redox-sensitive transcription factor NRF2. Moreover, we identify the histone acetyltransferase P300 as a redox-dependent modulator of BMAL1/CLOCK transactivation ability.

## Results

### Inhibition of the PPP Alters Circadian Redox and Transcriptional Oscillations

In red blood cells, peroxiredoxin oxidation rhythms resonate with NADPH oscillations (O’Neill and Reddy, 2011). NADPH powers intracellular redox defense and is used by the peroxiredoxin system during its catalytic cycle to remove harmful reactive oxygen species (Wood et al., 2003). We therefore measured NADPH accumulation in human osteosarcoma (U2OS) cells, an established and robust cellular clock model (Liu et al., 2008), and found similar redox oscillations to those seen in red blood cells previously (O’Neill and Reddy, 2011) (Figure 1B).

Given that the PPP is a major source of NADPH in the cell (Fan et al., 2014), we hypothesized that inhibiting its metabolic flux would affect redox oscillations. To disrupt NADPH production, we used 6-aminonicotinamide (6AN). This compound is metabolized into an analog of NADP^+^, thus competitively inhibiting the critical NADPH-producing enzymes 6-phosphogluconate dehydrogenase (PGD) and glucose 6-phosphate dehydrogenase (G6PD) (Köhler et al., 1970). Consistent with our hypothesis, 6AN treatment prolonged the period of NADPH oscillation to ∼30 hr (Figure 1B). We next measured peroxiredoxin oxidation in U2OS cells and found that these rhythms were similarly affected by inhibition of the PPP (Figures 1C, 1D, S1A, and S1B, available online), indicating that the availability of NADPH regulates circadian redox oscillations. 6AN treatment indeed drove the NADP^+^:NADPH redox poise in favor of oxidation by decreasing NADPH by ∼50%, consistent with its expected effect (Figure 1E). In contrast, NAD^+^:NADH ratio remained unchanged (Figure S1C) and treatment with 6AN did not acutely affect glycolysis or mitochondrial respiration rates (Figures S1D and S1E).

We then investigated the effect of PPP inhibition on transcriptional oscillations using U2OS cells stably expressing the *Bmal1*:*luciferase* (*Bmal1*:*luc*) reporter construct (Liu et al., 2008). Treatment of *Bmal1*:*luc* cells with 6AN caused a strong and reversible effect on transcriptional oscillations. Oscillation period was lengthened by 3 hr (Figures 1F, 1G, and S1F; 5 mM 6AN, 28.48 ± 0.16 hr versus control, 25.50 ± 0.05 hr), and after 96 hr of treatment, removal of the drug restored almost normal oscillations (5 mM 6AN, 25.65 ± 0.10 hr versus control, 24.97 ± 0.21 hr). We further validated this effect by using a genetic approach to repress PPP activity. We used RNA interference to knock down expression of the NADPH-producing enzymes G6PD and PGD in *Bmal1*:*luc* cells (Figures S2A–S2F). In agreement with pharmacological manipulations, we observed an increase in the NADP^+^:NADPH redox ratio (Figure S2D) and a significant period lengthening for both genes (Figure S2E).

Having shown that tonic inhibition of the PPP modulated the period of redox and transcriptional oscillations, we tested whether such metabolic perturbation could also reset the phase of circadian oscillations in a time-of-day-dependent manner. To this end, we administered 6AN treatment around the clock and assessed the phase of oscillations following treatment, generating a “phase-response curve.” Inhibiting the PPP had a strong resetting effect, inducing large phase advances or delays in rhythms depending on the time of day when the treatment started (Figures 1H–1J). Together, these results implicate the PPP as a regulator of two key facets of circadian pacemaker function (period and phase of oscillation).

### The PPP Affects Circadian Oscillations via NADPH Metabolism

**Figure 2:**
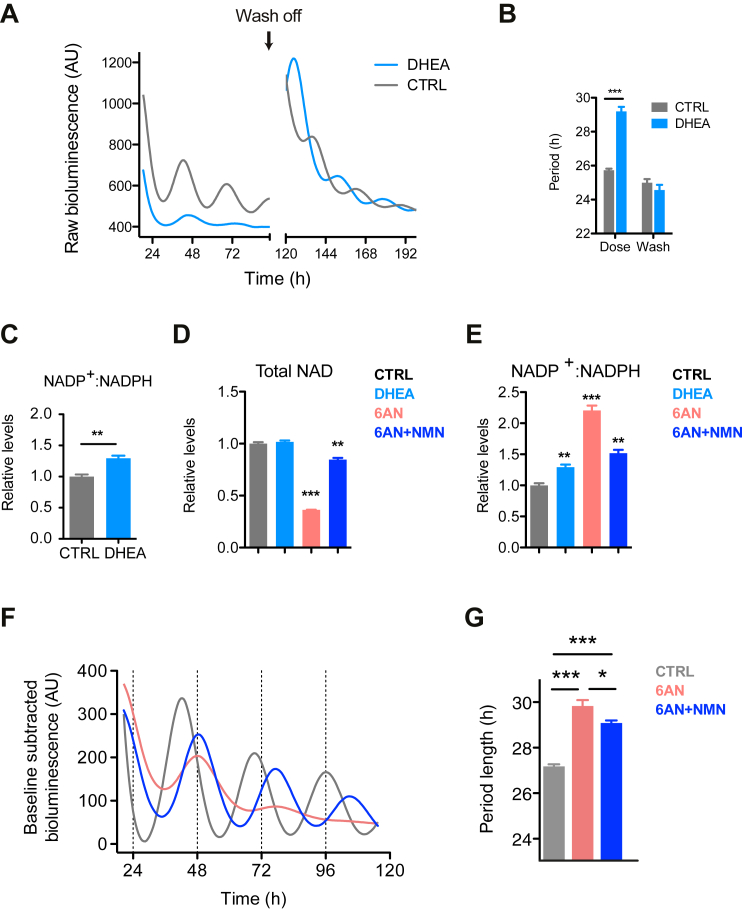
Manipulation of the PPP Affects Circadian Oscillations through NADPH (A) Bioluminescence traces for *Bmal1*:*luc* U2OS cells treated with 50 μM dehydroepiandrosterone (DHEA) versus control (DMSO), followed by wash off after 96 hr. (mean values shown, n = 3–6) (B) Quantifications of the period length from (A) before and after wash off (mean ± SEM, n = 3–6; two-tailed Student’s t test; ^∗∗∗^p < 0.001). (C) NADP^+^:NADPH ratio of cells treated with DHEA (mean ± SEM, n = 3–4; two-tailed Student’s t test; ^∗∗^p < 0.01). (D) Treatment of *Bmal1*:*luc* U2OS cells with 5 mM 6AN decreases the levels of total NAD, while incubation with 50 μM DHEA has no effect. NAD levels in presence of 6AN can be restored by addition of 500 μM NMN (control [DMSO] versus treated cells; mean ± SEM, n = 3–4; two-tailed Student’s t test; ^∗∗∗^p < 0.001, ^∗∗^p < 0.01). (E) Treatment with 500 μM NMN does not restore NADP^+^:NADPH ratio to normal levels (two-tailed Student’s t test, control [DMSO] versus treated cells; mean ± SEM, n = 3–4, ^∗∗∗^p < 0.001, ^∗∗^p < 0.01). (F) Bioluminescence traces for *Bmal1*:*luc* U2OS cells treated with 6AN, or 6AN and NMN, versus control (DMSO) (mean values shown, n = 8). (G) Quantifications of the period length from (F) (two-tailed Student’s t test; mean ± SEM, n = 8, ^∗∗∗^p < 0.001, ^∗^p < 0.05).

Since there is cellular interconversion of NAD^+^ and its phosphorylated form (NADP^+^), we next investigated if the effects of PPP perturbation could involve this pathway. This is important because NAD^+^ metabolism forms a feedback loop with the core circuitry of the circadian transcriptional network (Nakahata et al., 2009, Ramsey et al., 2009), and therefore changes in NAD^+^ might potentially contribute to the period phenotype seen with PPP inhibition. Therefore, we tested the effect of dehydroepiandrosterone (DHEA), a non-competitive inhibitor of G6PD (Raineri and Levy, 1970), and again found a reversible period lengthening (Figures 2A, 2B, and S3A–S3D) and an increase in the NADP^+^:NADPH redox ratio (Figure 2C). In contrast to 6AN treatment, DHEA did not affect the accumulation of total NAD (Figure 2D), showing that the effect on period does not depend on NAD levels and is specific to the change in NADP^+^:NADPH redox ratio. Importantly, we also determined conditions under which we could rescue NAD^+^ levels in 6AN-treated cells, using nicotinamide mononucleotide (NMN), a precursor of NAD^+^ (Figures 2D and 2E). When NAD levels were restored close to normal with NMN in the presence of 6AN, there was a rescue of the amplitude of oscillations, but the effect on period was not abolished (Figures 2F, 2G, and S3E). This therefore suggested that inhibition of the PPP differentially affects circadian oscillations through direct and indirect redox mechanisms, and that the prolonged period is specific to NADPH metabolism.

In addition to its redox role, the PPP is a key contributor to biosynthetic function, especially for nucleic acid synthesis. In order to globally assess the effect of inhibition by 6AN or DHEA, we performed metabolomics profiling of U2OS cells treated with the PPP inhibitors (Figure S3F; Table S3). We observed only mild perturbations in the levels of 90 metabolites, and the changes correlated between the 6AN and DHEA treatments (Figure S3G). Although the levels of ribose-5-phosphate (R5P), which is important for nucleotide synthesis, were slightly decreased in the both conditions, the levels of nucleotides and nucleosides were largely unchanged, indicating that the non-oxidative branch of the PPP, downstream of the NADPH-producing enzymes, was sufficient to provide substrates for synthetic pathways. Thus, the effects of both 6AN and DHEA appear specific to the oxidative (NADPH-producing) branch of the PPP, without a significant impact on its biosynthetic functions.

### Remodeling of Circadian Gene Expression by NADPH Metabolism

**Figure 3:**
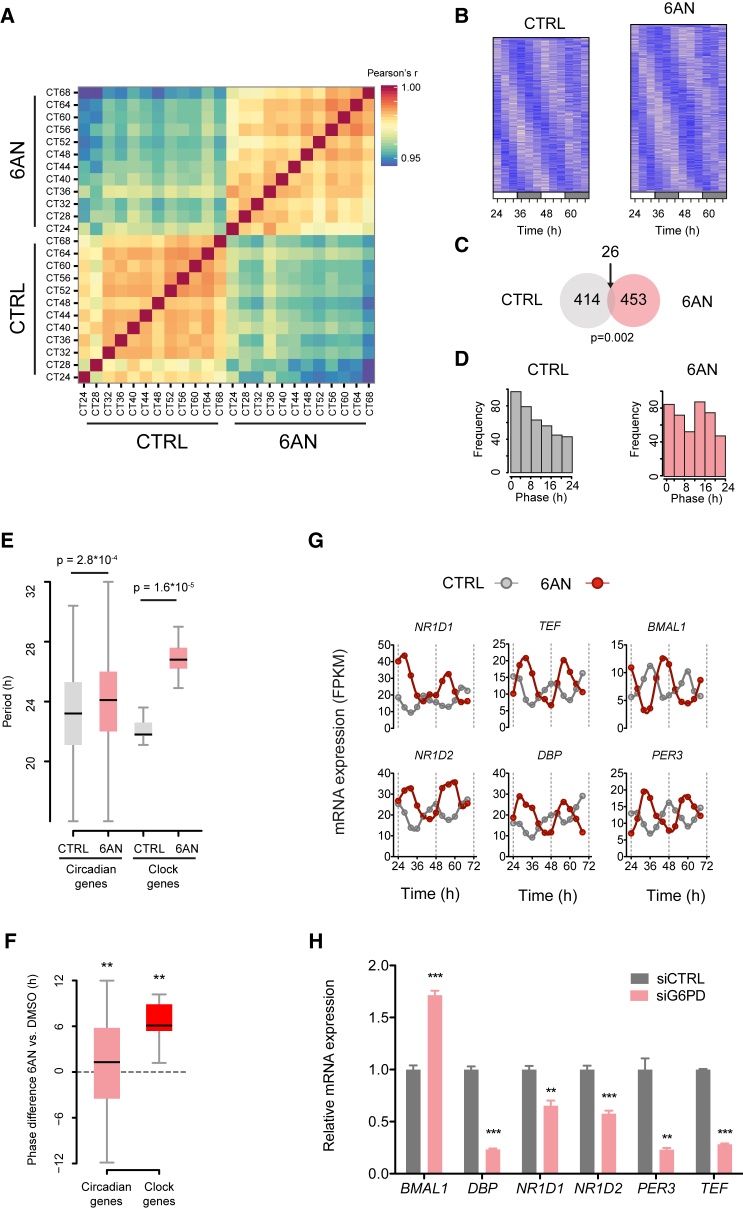
PPP Inhibition Remodels Circadian Gene Expression (A) Time course of mRNA expression determined by RNA-seq in *Bmal1*:*luc* U2OS cells incubated with 5 mM 6AN or control (DMSO). The heatmap shows the Pearson’s correlation coefficient between time points for log-transformed fragments per kilobase of transcript per million (FPKM) of the 14,686 expressed transcripts. *Bmal1*:*luc* cells were synchronized with a dexamethasone shock and total RNA was collected at the indicated time points. (B) Heatmap representation of the temporal accumulation of mRNA for circadian transcripts in the 6AN (453) and control (414) conditions. The RAIN algorithm (Thaben and Westermark, 2014) was used to detect circadian transcripts (p ≤ 0.01) in each dataset. (C) Overlap between the rhythmic transcripts detected in the 6AN and control conditions (Fisher test on contingency table, p = 0.002). (D) Phase histogram of rhythmic transcripts shown in (B). (E) Boxplot representation of period length for 6AN and control mRNA profiles for circadian transcripts (RAIN algorithm, p ≤ 0.01) and clock gene transcripts (list of 20 well-described circadian genes; Table S4) (Wilcoxon rank-sum test, with p values as shown). (F) Boxplot representation of phase differences between 6AN and control mRNA profiles for circadian transcripts (detected in the 6AN or control condition with RAIN algorithm, p ≤ 0.01) and clock gene transcripts (Kuiper’s one-sample test of uniformity; ^∗∗^p < 0.01). (G) Profiles of mRNA accumulations for the six clock genes that are detected as circadian in both conditions. (H) mRNA accumulation of clock gene transcripts in *Bmal1*:*luc* U2OS cells following siRNA knockdown with 50 nM *G6PD* or control siRNA (negative control #1) (mean ± SEM, n = 3; two-tailed Student’s t test; ^∗∗∗^p < 0.001, ^∗∗^p < 0.01).

How are perturbations in redox oscillations transduced into alterations in circadian gene expression and, ultimately, to organism behavior? To probe this, we performed time course analyses of U2OS cells and determined their gene expression profiles by RNA sequencing (RNA-seq) (Figures 3A and S4A). 6AN treatment did not globally affect the transcriptome, as we observed high correlation between control and 6AN-treated samples (Pearson correlation coefficient >0.94 between all time points; Figure 3A). However, perturbation of NADPH metabolism caused a profound change in circadian gene expression (Figures 3B–3D). Using the RAIN algorithm (Thaben and Westermark, 2014), we detected 414 and 453 circadian transcripts in the control and 6AN condition, respectively (Figure 3B), with 26 common transcripts (Figure 3C). We validated these analyses using two other algorithms, Fisher test (Rey et al., 2011) and ARSER (Yang and Su, 2010), and found a considerable overlap, as 147 and 169 genes were detected by the three methods in the control and 6AN condition, respectively (Figure S4B). Gene ontology (GO) analysis of rhythmic transcripts revealed that genes involved in metabolic processes were enriched in both conditions, while GO annotations related to circadian rhythms were highly enriched in the 6AN condition (Figure S4C). Our results thus indicate that perturbation of the PPP was able to extensively remodel circadian gene expression, as highlighted by the altered phase distribution of mRNA expression (Figure 3D).

This led us to investigate further how the period and phase of circadian transcripts were changed following PPP inhibition. We found that the median period of oscillations was increased by treatment with 6AN (Figure 3E; control versus 6AN, 23.2 hr versus 24.1 hr). Interestingly, the effect on clock genes was especially pronounced (control versus 6AN, 21.8 hr versus 27.2 hr) but only marginally contributed to the shift in the period distributions of all circadian transcripts (Figure S4D). Similarly, we computed the distribution of phase differences between 6AN and control conditions. We observed a phase delay for both circadian and clock gene sets (Figures 3F and S4E), again with a stronger effect on clock genes. Accordingly, *NR1D1*, *NR1D2*, *TEF*, *DBP*, *BMAL1*, and *PER3*, the six clock genes that are rhythmic in both conditions, displayed prolonged periods and phase delays in their mRNA accumulation profiles (Figure 3G). Most other clock genes (Table S4) had similar effects on circadian gene expression, even if they were not necessarily detected as statistically rhythmic (Figure S4F). In order to validate the effect of PPP inhibition on circadian gene expression, we silenced the expression of the enzyme G6PD by small interfering RNA (siRNA) knockdown. In agreement with treatment with 6AN, we observed a perturbation of the circadian gene network, since the expression of clock genes was severely disrupted (Figure 3H). Therefore, inhibition of the PPP remodels circadian expression by changing the period and phase of circadian transcripts, with an effect especially prominent on clock genes.

### The Circadian Transcription Factors BMAL1 and CLOCK Are Activated by a Change in Redox Environment

**Figure 4:**
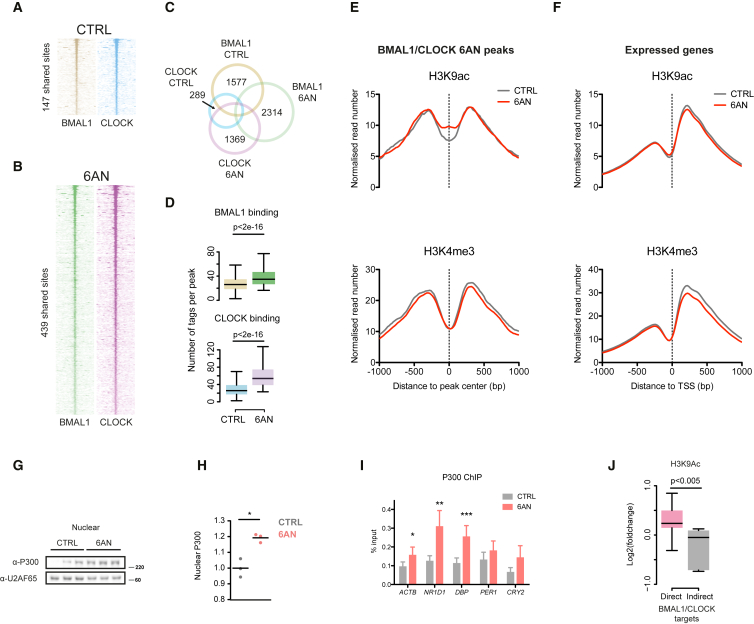
BMAL1/CLOCK Are Activated by Inhibition of the PPP (A and B) BMAL1 and CLOCK ChIP-seq binding profiles around the 147 and 439 BMAL1/CLOCK peaks bound, respectively, in the control (A) and 6AN conditions (B). *Bmal1*:*luc* U2OS cells were treated with 5 mM 6AN or control (DMSO) and chromatin was extracted after 24 hr of incubation. (C) Venn diagram showing the overlap of ChIP-seq peaks for BMAL1 and CLOCK. The total number of peaks for each set is given. (D) Distributions of number of tags per peak in the 439 peaks shared between 6AN BMAL1 and CLOCK ChIP-seq for BMAL1 (top) and CLOCK (bottom) (Kolmogorov-Smirnov test performed between the indicated distributions, with p values as shown). (E and F) Genomic profiles of H3K9ac (top) and H3K4me3 (bottom) densities around BMAL1/CLOCK 6AN peaks (E) and transcription start sites (TSSs) of the 14,686 expressed transcripts (F). (G) Immunoblot showing P300 nuclear accumulation in cells treated with 6AN or control (DMSO). U2AF65 is shown as loading control. Molecular weights (kDa) shown on right side of blots. (H) Densitometric quantification of blots from (G) (two-tailed Student’s t test, ^∗^p < 0.05). (I) ChIP followed by quantitative real-time PCR of P300 following 6AN treatment or control (DMSO) (mean ± SEM, n = 3; two-tailed Student’s t test, ^∗^p < 0.05). (J) Distribution of fold changes in H3K9ac density (6AN versus control) for direct and indirect BMAL1/CLOCK target genes (Wilcoxon rank-sum test, with p values as shown).

Since the core circadian transcription factors BMAL1 and CLOCK regulate the expression of most of the clock genes, we hypothesized that perturbation in circadian gene expression may involve a change in BMAL1/CLOCK DNA-binding activity. This response could indeed result from an altered NADP^+^:NADPH (or NAD^+^:NADH) ratio, since these dinucleotides have been reported to affect the binding affinity of several circadian PAS-domain transcription factors in vitro (Rutter et al., 2001). We therefore performed chromatin immunoprecipitation followed by sequencing (ChIP-seq) in *Bmal1*:*luc* U2OS cells to delineate genome-wide binding patterns of these transcription factors. We found that the number of shared BMAL1/CLOCK genomic binding sites increased from 147 to 439 (3-fold increase) following 6AN treatment (Figures 4A and 4B). CLOCK was mostly affected, since we observed a more than 4-fold rise in genomic binding peaks (Figure 4C). Moreover, BMAL1 and CLOCK binding strengths significantly increased at 439 shared peaks following PPP inhibition (Figure 4D), indicating enhanced DNA-binding activity that is consistent with elevated expression of several BMAL1/CLOCK targets upon 6AN treatment (Figures 3G and S4F).

Increased DNA-binding activity of BMAL1/CLOCK was accompanied by changes in chromatin state at their genomic binding sites (Figures S5A and S5B). We measured two epigenetic marks of transcriptionally active chromatin by ChIP-seq: histone H3 lysine 9 acetylation (H3K9ac) and histone H3 lysine 4 trimethylation (H3K4me3) (Figures 4E and 4F). While H3K4me3 profiles remained unchanged, H3K9 showed a local increase around BMAL1/CLOCK sites (Figure 4E). This effect was not due to a widespread increase in H3K9 acetylation near active promoters, as H3K9ac profiles around transcription start sites (TSSs) of expressed genes were not affected (Figure 4F). Moreover, elevated H3K9 acetylation was specific to 6AN peaks, as we did not observe similar effects at BMAL1/CLOCK peaks from the control condition (Figure S5C). Notably, BMAL1/CLOCK binding and H3K9 acetylation were associated with rhythmic expression of nearby transcripts. Indeed, we found that the fraction of rhythmic transcripts increased with the fold change in BMAL1/CLOCK binding and H3K9 acetylation following 6AN treatment (Figures S5D and S5E).

We next investigated the mechanism by which redox imbalance could affect chromatin states. First, we excluded the NAD^+^-dependent deacetylase SIRT1 as a mechanism driving this change, since *SIRT1*^–/–^ mouse embryonic fibroblasts exposed to 6AN still exhibited alterations in clock gene mRNA patterning (Figure S5F). Moreover, rescue of NAD^+^ levels with NMN did not restore normal DNA-binding activity of BMAL1/CLOCK or levels of H3K9ac (Figure S5G). These results indicate that SIRT1 and other NAD^+^-dependent deacetylases, including SIRT6, are not likely to significantly contribute to the chromatin state changes we saw with redox perturbation. Therefore, we hypothesized that the archetypal histone acetyltransferase P300 might mediate these effects, since it is able to form disulphide bridges with the FOXO transcription factors by a redox-dependent mechanism (Dansen et al., 2009) and has been shown to interact with clock proteins (Etchegaray et al., 2003). We measured P300 protein accumulation in the nucleus and observed increased levels following 6AN treatment (Figures 4G and 4H). Furthermore, ChIP analyses revealed that clock gene loci exhibited elevated P300 binding upon PPP inhibition (Figure 4I), strongly implicating redox-dependent acetylation by P300 at these genomic regions. Interestingly, increased P300 binding and H3K9ac were specific to direct BMAL1/CLOCK targets—those with mRNA expression in phase with DNA-binding activity (Rey et al., 2011) (Figures 4I and 4J; Table S4). Furthermore, we investigated if *P300* knockdown by siRNA could antagonize the effect of 6AN on circadian oscillations. Consistent with its role in activating circadian transcription, *P300* knockdown caused a strong decrease of the amplitude of circadian oscillations (Figure S5H). However, at low siRNA concentrations, we found that *P300* knockdown was able to partially reverse the strong period lengthening effect of 6AN, as it reduced the period difference to only 1 hr compared to control (Figure S5I). These results thus indicate that PPP inhibition leads to a redox-dependent activation of BMAL1/CLOCK that is mediated by the histone acetyltransferase P300.

### NRF2 Signaling Links Changes in Redox Balance to Circadian Gene Expression

**Figure 5:**
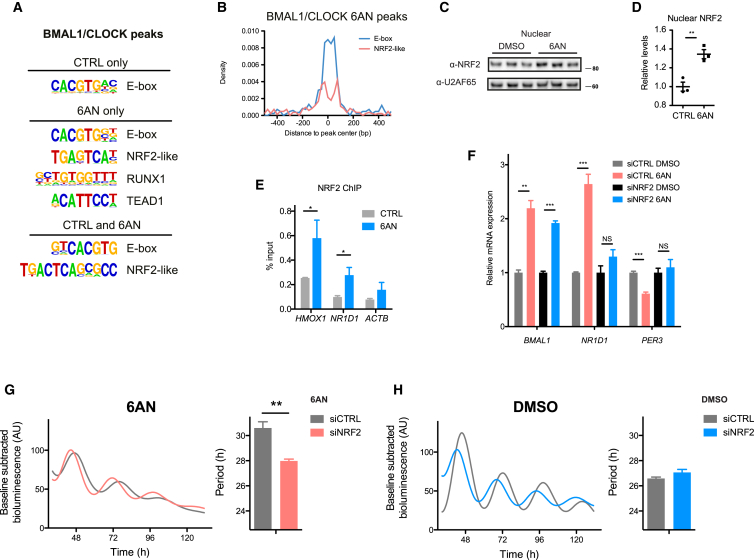
NRF2 Mediates the Effect of Redox Perturbation on Circadian Oscillations (A) De novo HOMER motif analysis of the indicated sets of BMAL1/CLOCK peaks. (B) Histogram of E-box and NRF2-like motif positions around BMAL1/CLOCK peaks bound only the 6AN condition. (C) Immunoblot showing NRF2 nuclear accumulation in cells treated with 6AN or control (DMSO). U2AF65 is shown as loading control. Molecular weights (kDa) shown on right side of blots. (D) Densitometric quantification of blots from (C) (mean ± SEM, n = 3; two-tailed Student’s t test, ^∗∗^p < 0.01). (E) ChIP followed by quantitative real-time PCR of NRF2 following 6AN treatment or control (DMSO) (mean ± SEM, n = 3; one-tailed Student’s t test, ^∗^p < 0.05). *HMOX1*, heme oxygenase 1. (F) mRNA accumulation of clock gene transcripts in *Bmal1*:*luc* U2OS cells following knockdown with 20 nM *NRF2* siRNA or control (non-targeting siRNA #1). *Bmal1*:*luc* cells were synchronized 72 hr after transfection with a dexamethasone shock, and total RNA was collected after 24 hr incubation with 5 mM 6AN or control (DMSO) (mean ± SEM, n = 3; two-tailed Student’s t test; ^∗∗∗^p < 0.001, ^∗∗^p < 0.01). NS, not statistically significant by t test. (G and H) Bioluminescence recordings of *Bmal1*:*luc* U2OS cells transfected with 20 nM *NRF2* siRNA or control (non-targeting siRNA #1) combined with 6AN treatment at 1.25 mM (G) or control (DMSO) (H) (left; mean, n = 8). Quantifications of circadian period length of bioluminescence traces (right; mean ± SEM, n = 8; two-tailed Student’s t test; ^∗∗^p < 0.01).

Overlap between circadian and BMAL1/CLOCK-bound genes was significant for the control, but not the 6AN, condition (Fisher test; control, p < 1 × 10^−3^; 6AN, p = 0.05; Figure S6A), suggesting that additional transcription factors were likely to contribute to the remodeling of circadian gene expression. Consistent with this observation, we found enriched DNA motifs for other transcription factors in the 6AN condition (Figure 5A) and, in particular, a motif corresponding to the redox-sensitive transcription factor NRF2 (Chorley et al., 2012) (Figure S6B). NRF2-like motifs showed a positional correlation with the canonical BMAL1/CLOCK binding motif (E-box) when 6AN-treated cells were assessed (Figure 5B). Importantly, we observed a significant increase in NRF2 nuclear accumulation following 6AN treatment (Figures 5C and 5D), indicating that PPP inhibition leads to the activation of NRF2. H3K9ac genomic profiles around NRF2 ChIP-seq peaks (Chorley et al., 2012) were not altered by 6AN treatment, indicating that NRF2 activation is not associated with H3K9 acetylation (Figure S6C).

We next investigated whether *NRF2* could mediate the interaction between redox balance and circadian oscillations. First, we observed that a significant fraction of circadian transcripts in the control and 6AN conditions were NRF2 targets (44 and 48 genes, respectively), implicating this redox transcription factor in the control of circadian gene expression (Figures S6D and S6E). Two important NRF2 targets, glutathione reductase (*GSR*) and thioredoxin reductase 1 (*TXNRD1*), which both use NADPH as reducing agent for cellular redox defense, also displayed rhythmic mRNA accumulation, even though they were not statistically detected as circadian (Figure S6F). Importantly, the circadian transcriptional repressor *NR1D1* was among NRF2 targets, with inducible binding sites at its promoter and in its first intron (Figure S6G) (Chorley et al., 2012). Accordingly, we found that perturbation of the PPP caused an increase in NRF2 DNA binding to *NR1D1* and its known target gene heme oxygenase 1 (*HMOX1*) (Figure 5E), suggesting that the PPP-dependent activation of NRF2 could relay redox signals to the circadian network through *NR1D1*.

In order to functionally validate the role of *NRF2* in mediating the effect of 6AN on circadian gene expression, we silenced *NRF2* expression using siRNA and measured the mRNA expression of several clock genes (Figures 5F and S6H). We found that both *NR1D1* and *PER3* lost their responsiveness to 6AN treatment when *NRF2* was silenced, indicating that *NRF2* knockdown can reverse the effects caused by inhibition of the PPP. Bioluminescence recordings of *Bmal1*:luc U2OS cells confirmed this hypothesis, since *NRF2* silencing in 6AN-treated cells reduced the period by 2 hr compared to control siRNA (Figure 5G; siCTRL, 30.6 ± 0.5 hr; siNRF2, 28.0 ± 0.2 hr). In contrast, *NRF2* silencing in control cells did not affect the period of oscillations (Figure 5H; siCTRL, 26.6 ± 0.1 hr; siNRF2, 27.1 ± 0.2 hr). We further validated this effect using DHEA and found again that *NRF2* silencing reversed the period lengthening caused by inhibition of the PPP (Figures S6I and S6J). Our results thus identify NRF2 as a key connection between redox and circadian oscillations.

### Inhibition of the PPP Modulates Circadian Oscillations in Mouse Tissues

**Figure 6:**
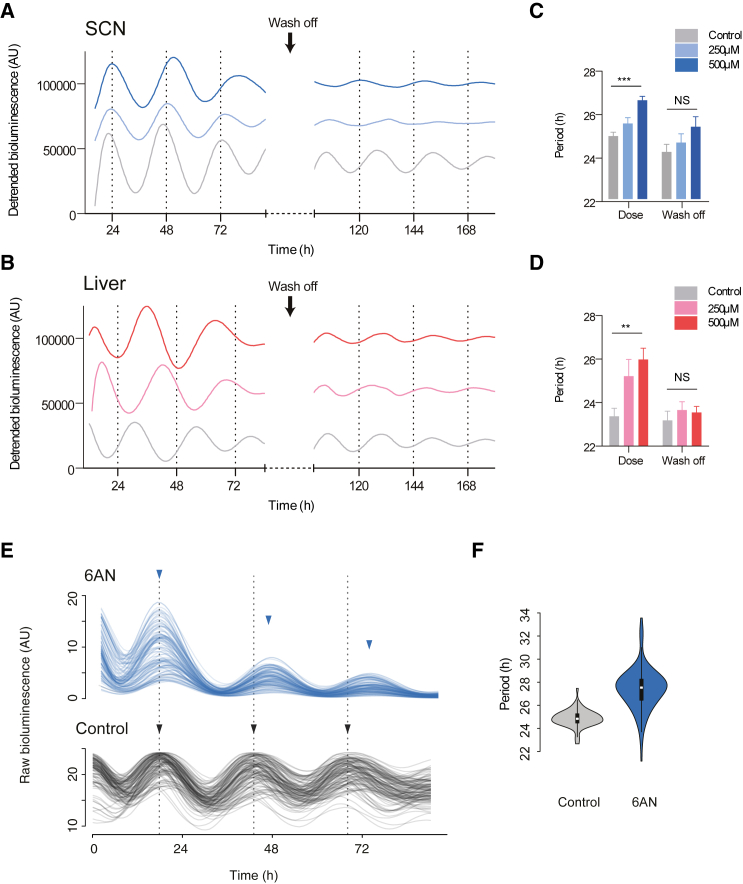
PPP Perturbation Disrupts Circadian Oscillations in Mouse Tissues (A and B) Bioluminescence recordings of suprachiasmatic nuclei (SCN) (A) and liver slices (B) from *mPer2*^*Luciferase*^ (*mPer2*^*Luc*^) mice treated with the indicated concentration of 6AN or control (DMSO) (left), followed by wash off (right) with control medium. (C and D) Quantifications of the period length from SCN (C) and liver (D) slices treated with 6AN or control (DMSO) before and after wash off (mean ± SEM, n = 3–7; two-tailed Student’s t test; 500 μM versus control; ^∗∗∗^p < 0.001, ^∗∗^p < 0.01). NS, not statistically significant by t test. (E) Single-cell bioluminescence traces of SCN slices from *mPer2*^*Luc*^ mice incubated either with 500 μM 6AN or control (DMSO) (n > 100). (F) Violin plot representing the difference in the distribution of circadian period lengths in the 6AN and control (DMSO) conditions (n > 100; two-tailed Student’s t test, p < 1 × 10^−16^).

Having explored how inhibition of the PPP leads to altered circadian gene expression, we set out to study the effect of these metabolic perturbations on circadian behavior, an important output of the clockwork. Destruction of erythrocytes (hemolysis) when the 6AN target G6PD is deficient (Cappellini and Fiorelli, 2008) indicated that an in vivo approach in live mice would not yield meaningful results. We therefore next analyzed the effects of PPP inhibition on circadian rhythmicity in primary tissues ex vivo. Treatment of organotypic slice cultures of the suprachiasmatic nucleus (SCN) and a key metabolic tissue, the liver, from *mPer2*^*Luciferase*^ (*mPer2*^*Luc*^) mice (Yoo et al., 2004) with 6AN elicited a period lengthening at the highest concentration (500 μM 6AN; SCN, 26.6 ± 0.2 hr; liver, 26.0 ± 0.5 hr versus control SCN, 24.9 ± 0.2 hr and liver, 23.4 ± 0.4 hr), similar to the effect in human cells (Figures 6A–6D, S7A, and S7B). Importantly, removal of 6AN after 4 days of incubation restored a normal period length in both tissues, illustrating the reversible nature of the redox perturbations.

Given that an ensemble of oscillators may exhibit comparable behavior due to dispersion in the phase of individual oscillators within the tissue, we performed single-cell imaging of *mPer2*^*Luc*^ SCN slices to investigate the effect on period length in individual cells. Similar to the effects at the population level, single cells exhibited a period lengthening of >2 hr when treated with 6AN (500 μM 6AN, 27.5 ± 0.2 hr versus control, 25.0 ± 0.1 hr) (Figures 6E and S7C), together with a higher damping rate (Figure S7D). Notably, we obtained similar results when measuring single-cell oscillations in *Per2*:*luc* U2OS cells treated with 6AN (Figures S7E–S7G), and we did not observe deleterious changes in cell morphology upon exposure to 6AN or DHEA for several days (Figure S7H). This highlights the significant and specific effect that perturbing central cellular metabolism has on cell-autonomous circadian oscillations.

### Fly Behavioral Rhythms Respond to Redox Perturbation Similarly to Mammals

**Figure 7:**
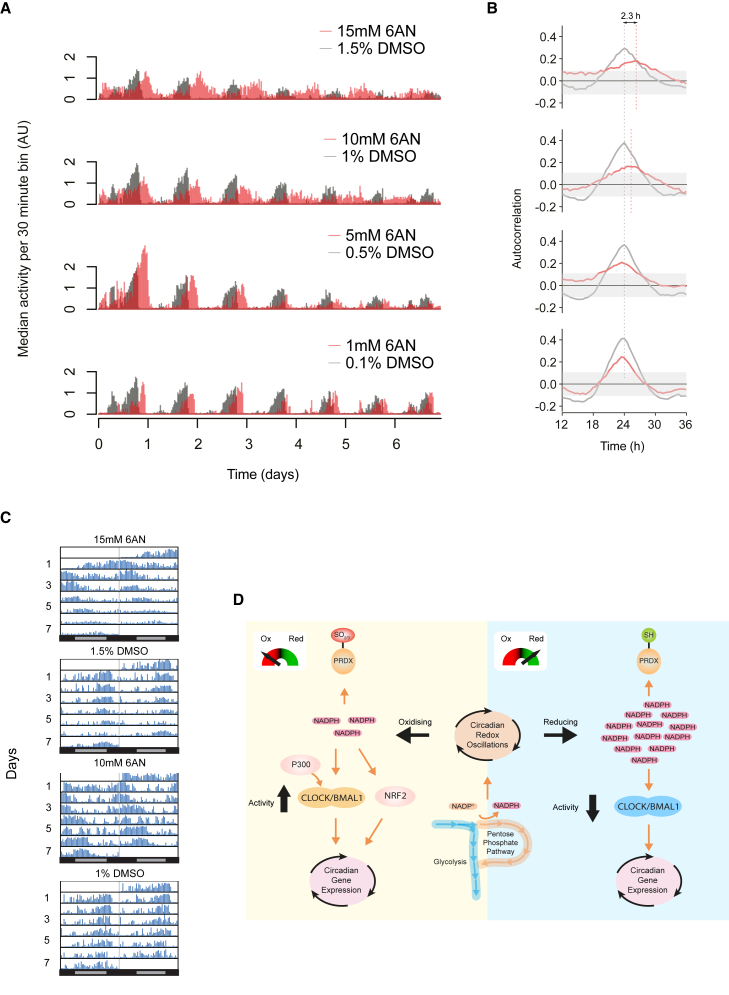
PPP Inhibition Affects Behavioral Rhythms in Flies (A) Median activity plots of *Drosophila melanogaster* (Canton-S strain) behavioral activity with concentrations of 6AN ranging from 1 to 15 mM in their usual growth medium. As a control, DMSO was used at the specified concentration to directly match the concentration experienced with the 6AN dose. (B) Mean autocorrelation of activity plots highlights the period difference between 6AN and DMSO conditions. The 95% confidence interval (white noise) is shown as a shaded gray area and autocorrelation values outside these boxes are significant at p < 0.05 (n = 24 male flies per group). (C) Representative actograms of individual flies following treatment with the indicated concentration of 6AN or control (DMSO). (D) Schematic showing how perturbation of the PPP regulates circadian redox and transcriptional oscillations.

In order to circumvent the systemic effects associated with 6AN in mice, we measured the effect of PPP inhibition on *Drosophila melanogaster* locomotor activity. Flies are ideal organisms to study the effect of metabolic inhibitors because they lack red blood cells, which are sensitive to such treatment when administered to rodents. In addition, their transcriptional clockwork is largely similar in architecture to mammals, with orthologs of BMAL1 and CLOCK driving gene expression of repressors (Young and Kay, 2001). We therefore recorded locomotor behavior of flies using a video recording system (Figure S7I). Behavioral recordings of flies fed 1–15 mM 6AN in their agar growth medium revealed a dose-dependent effect on behavioral rhythms (Figure 7A). PPP inhibition caused only a mild reduction in the amplitude of activity rhythms at all concentrations, enabling us to measure their behavioral rhythms over the course of several days. Treatment with 6AN lengthened the period up to 2.3 hr at the highest dose (15 mM) (Figure 7B), and this effect was visible in the locomotor activity of individual flies (Figures 7C and S7J). These results thus show that inhibition of the PPP not only affects cell-autonomous circadian oscillations but also complex behavioral rhythms controlled by the circadian clock.

## Discussion

Models for circadian timekeeping in all species currently incorporate similar transcriptional mechanisms. However, each species’ clock relies on a different set of clock genes in its timing system, given that these are not evolutionarily conserved between kingdoms (Young and Kay, 2001). Recently, an alternative type of circadian oscillation, the oxidation of peroxiredoxins, has been reported in a diverse range of species (Edgar et al., 2012), implying that redox oscillations could be a more fundamental timekeeping mechanism. We set out to investigate how such non-transcriptional oscillations may be connected to circadian transcriptional rhythms. Our results demonstrate that manipulation of the PPP, a key pathway in NADPH metabolism, affects circadian oscillations in human cells, mouse tissues, and living flies. Identification of the PPP as a modulator of redox oscillations indicates that the overoxidation pattern of peroxiredoxin may be a reporter of more fundamental oscillations in the form of NADPH rhythms (Figure 7D). It also suggests that NADPH metabolism may be an important parameter in the generation of circadian redox oscillations, in light of previous findings showing similar NADPH rhythmicity in non-transcriptional models (O’Neill and Reddy, 2011). Moreover, these redox rhythms may have physiological importance, since several studies have described rhythms in NADP^+^:NADPH ratio in rodents (Reddy and Rey, 2014).

We found that redox perturbations increased the DNA-binding activity of BMAL1/CLOCK, which in turn led to profound qualitative and quantitative changes in circadian gene expression. The effects were especially prominent for clock genes, but inhibition of the PPP also caused a switch in the set of output circadian genes. Indeed, the sets of genes being rhythmic with or without PPP inhibition diverged considerably. Perturbation of NADPH metabolism also led to an increased density of histone H3K9 acetylation near BMAL1/CLOCK sites, indicating redox-dependent chromatin remodeling. We showed that the redox-sensitive histone acetyltransferase P300 accumulated in the nucleus after PPP inhibition and subsequently displayed increased binding at BMAL1/CLOCK sites. Interestingly, this effect was more pronounced at direct BMAL1/CLOCK target genes—those with mRNA expression corresponding to BMAL1/CLOCK binding in mouse liver (Rey et al., 2011)—suggesting that P300 is mainly associated with transcriptionally active BMAL1/CLOCK complexes. This is consistent with the fact that the genome-wide binding of P300 is in phase with BMAL1/CLOCK binding in mouse liver (Koike et al., 2012). Thus, our study indicates that P300 links redox rhythms to circadian transcription by modulating BMAL1/CLOCK transactivation ability in a redox-dependent fashion.

Our study also revealed the important role of *NRF2* in the interplay between redox and circadian oscillations. Previous studies have shown clock-controlled activity of *Nrf2* in the mouse lung (Pekovic-Vaughan et al., 2014) and proposed that *Nr1d1* may respond to oxidative stress signals through an NRF2 binding site in its promoter (Yang et al., 2014). Here we find that NRF2 and BMAL1/CLOCK have overlapping transcriptional regulatory programs, likely through cooperative binding to common genomic sites, and may therefore contribute to circadian transcription, as suggested by the number of NRF2 target genes rhythmically expressed. Moreover, our data strengthen the notion that *NR1D1* could integrate circadian and redox signals, but most importantly reveal the role of *NRF2* as an important regulatory node between redox rhythms and circadian transcriptional oscillations in nucleated cells. Indeed, we found that *NRF2* is necessary for relaying redox perturbation caused by inhibition of the PPP to the circadian clockwork. These findings will be of great importance in building an integrated model of the circadian clock that encompasses its transcriptional and metabolic components. In addition, these results also provide a novel molecular mechanism by which redox imbalance, as experienced in cancer, cardiovascular disease, and neurodegenerative disease, could lead to circadian disruption.

In conclusion, we show that the PPP is an important regulator of circadian redox and transcriptional oscillations. We also identify P300 and NRF2 as two parallel mechanisms that connect redox oscillations to BMAL1/CLOCK-mediated transcriptional oscillations in nucleated cells. In a physiological context, the PPP is a fundamental player in anabolic cellular processes and is emerging as a determinant in cancer because of its role in curbing oxidative stress (Masri et al., 2015, Patra and Hay, 2014, Tsouko et al., 2014). Since the circadian transcriptional network rhythmically regulates over 40% of all protein-coding genes in the body (Zhang et al., 2014), an implication of our results is that disruption of metabolic pathways as occurs in many metabolic disorders and cancers could impact significantly on tissue gene expression programs and associated organ physiology via its effect on the clockwork.

## Experimental Procedures

### Cell Culture and Bioluminescence Assays

*Bmal1*:*luc* U2OS and *Per2*:*luc* U2OS cells were a gift from Dr. Andrew Liu, University of Memphis (Liu et al., 2008). U2OS cells were cultured in standard conditions. For bioluminescence recordings, U2OS cells were synchronized by changing medium to “Air Medium” (Hastings et al., 2005). Bioluminescence assays were performed at 37°C using 12-well and 96-well plates in custom-made bioluminescence recording systems (Cairn Research Ltd) composed of a charge-coupled device (CCD) camera (Andor iKon-M 934) mounted on the top of an Eppendorf Galaxy 170R CO_2_ incubator. Bioluminescence data traces were analyzed using a modified version of the R script “CellulaRhythm” (Hirota et al., 2008).

### Gel Electrophoresis and Immunoblotting

*Bmal1*:*luc* U2OS cells treated with 5 mM 6AN or control (DMSO) were synchronized with a dexamethasone shock and lysed in 1× SDS sample buffer at the indicated time points. NuPAGE Novex 10% Bis-Tris gradient gels were run according to the manufacturer’s protocol with a nonreducing MES SDS buffer system. Protein transfer to nitrocellulose for blotting was performed and membranes were incubated in anti-PRDX-SO3 (LF-PA0004, Thermo Fisher Scientific) or anti-ACTB (sc-47778, Santa Cruz) overnight at 4°C. Immunoblot signals were first normalized with loading control (actin) and then normalized to the average for each replicate.

### siRNA Transfections

For bioluminescence experiments, 90 μL cell suspension (0.5–1 × 10^5^ cells per mL) were seeded in 96-well plates. Cells were transfected with the indicated siRNAs (see Table S1 for details) 20–24 hr after seeding using Lipofectamine RNAiMAX (Life Technologies) according to manufacturer’s instructions. The medium of transfected cells was changed to “Air Medium” for bioluminescence recording 72 hr after transfection. When combined with drug experiments, solvent (DMSO) was kept at a concentration of 0.25% for control and treatment conditions. For gene expression analyses after siRNA knockdown, siRNA transfections were performed as described above, except that they were performed in 12-well plates, keeping the ratio between cell number and transfection reagent constant. Cells were synchronized with dexamethasone and cultured in DMEM supplemented with 5 mM 6AN or control (DMSO) 72 hr after transfection. After 24 hr incubation, RNA was extracted with TRI-Reagent in triplicate and purified with Direct-zol RNA MiniPrep kit (Zymo Research).

### RNA-Seq

For mRNA expression time course, *Bmal1*:*luc* U2OS cells were synchronized with dexamethasone (Figure S4A) and cultured in DMEM as described above, supplemented with 5 mM 6AN or a matched amount of DMSO (0.5%) as a control. At the time points indicated in the main text, RNA was extracted with TRI-Reagent in triplicate and purified with Direct-zol RNA MiniPrep kit (Zymo Research). RNA-seq libraries were prepared as described in the detailed protocol provided in Supplemental Experimental Procedures. Sequencing using a HiSeq platform with single-end 50 bp reads and subsequent quality filtering of reads was performed according to manufacturer’s instructions (Illumina).

### ChIP-Seq

ChIP was performed on *Bmal1*:*luc* U2OS using a modified version of an established protocol (Mortazavi et al., 2006) provided in Supplemental Experimental Procedures. ChIP-seq libraries were prepared as described for RNA-seq samples, except that fragment size selection was performed after end repair using AMPure XP Magnetic Beads. Sequencing using a HiSeq platform with paired-end 101 bp reads and subsequent quality filtering of reads was performed according to manufacturer’s instructions (Illumina).

### Nuclear Fractions

Nuclear fractions were prepared from *Bmal1*:*luc* U2OS cells treated with 6AN or control (DMSO) for 24 hr using the NE-PER reagents (Thermo Fisher Scientific) according to manufacturer instructions. Nuclear lysates were diluted with denaturing LDS sample buffer (Invitrogen) with 50 mM TCEP and heated to 70°C for 10 min before loading on gels. Nuclear extracts were analyzed by immunoblotting as described in the Supplemental Experimental Procedures, except that NuPAGE Novex 4%–12% Bis-Tris gradient gels were used. The following antibodies were used: anti-p300 (N-15), sc-584, Santa Cruz; anti-NFE2L2, Antibody EP1808Y, OriGene Technologies; and anti-U2AF65 U4758, Sigma.

### Organotypic Slice Culture and Bioluminescence

All animal experimentation was licensed by the UK Home Office under the Animals (Scientific Procedures) Act 1986, and according to the European Parliament and Council of the European Union Directive 2010/63/EU. Local Ethical Review was also conducted by the University of Cambridge. Prior to use in experiments, animals were group housed in individually ventilated cages under a 12:12 light:dark (LD) cycle with food and water available ad libitum. SCN and liver slices were extracted from 8- to 12-week-old adult *mPer2*^*Luc*^ mice (Yoo et al., 2004). Slices were cultured on a membrane (Merck Millipore, PICM0RG50) in a sealed dish. Slices were then transferred to custom-imaging incubators for whole-explant bioluminescence recording, or microscopes for single-cell bioluminescence imaging. Whole-explant imaging of SCN and liver slices was performed using an Andor iKon-M 934 cooled CCD camera mounted CO_2_ incubator at 37**°**C. Single-cell images were recorded from SCN slices placed into an Okolab stage-top heated chamber (37°C) mounted on an inverted Nikon Eclipse Ti-E microscope equipped with an electron-multiplied CCD (EM-CCD) camera (Hamamatsu ImagEM 1K, C9100-14).

### Fly Behavioral Assays

Wild-type Canton-S flies were bred and grown on standard yeast cornmeal agar medium at 25°C in 12 hr:12 hr LD cycles. For behavioral recording experiments, individual flies were placed into wells of a 96-well plate following brief exposure to CO_2_ anesthesia. Each well contained an equal volume of assay medium (5% sucrose, 1% agar), supplemented with 6AN or DMSO (control) at concentrations indicated in the main text. Although the concentrations of drug were high in comparison to those used in our cell and tissues studies, it is important to note that the *Drosophila* were ingesting agar dosed with the drug and therefore received a much lower effective concentration. Using a custom-made infrared video recording system, the locomotor activity of individual 4- to 7-day-old flies was recorded in constant darkness (DD) following 2 days of entrainment in LD cycles (which were not recorded). The videos were processed using Ethovision XT v10 software (Noldus) to quantify the locomotor activity of the flies.

## Author Contributions

A.B.R. and G.R. designed and planned experiments with contributions from J.S.O. G.R., K.A.F., N.B.M., U.K.V., and A.S. performed cell experiments. U.K.V. performed fruit fly experiments. G.R. and U.K.V. performed and analyzed RNA-seq and ChIP-seq experiments. V.V. supervised the metabolomics analyses and performed data analyses, and V.V. and G.R. analyzed results. L.W. and L.A.-B. performed mouse experiments. G.R., A.B.R., N.B.M., L.W., U.K.V., and V.V. analyzed the data. A.B.R. and G.R. wrote the manuscript with contributions from all of the authors.

## Accession Numbers

The GEO (http://www.ncbi.nlm.nih.gov/geo/) accession number for the RNA-seq and ChIP-seq data reported in this paper is GEO: GSE74439.

## Supplemental Information

Document S1. Supplemental Experimental Procedures, Figures S1–S7, and Tables S1–S4

Document S2. Article plus Supplemental Information
