# Supplementary material for: The Pentose Phosphate Pathway Regulates the Circadian Clock
Source: Cell Metab. 2016 Sep 13;24(3):462–73. doi: 10.1016/j.cmet.2016.07.024 (PMC5031559; doi:10.1016/j.cmet.2016.07.024)
Supplement: Document S1. Supplemental Experimental Procedures, Figures S1–S7, and Tables S1–S4 [file mmc1.pdf]

Cell Metabolism, Volume *24*

Supplemental Information

The Pentose Phosphate Pathway

Regulates the Circadian Clock

Guillaume Rey, Utham K. Valekunja, Kevin A. Feeney, Lisa Wulund, Nikolay B. Milev, Alessandra Stangherlin, Laura Ansel-Bollepalli, Vidya Velagapudi, John S. O'Neill, and Akhilesh B. Reddy

Figure S1

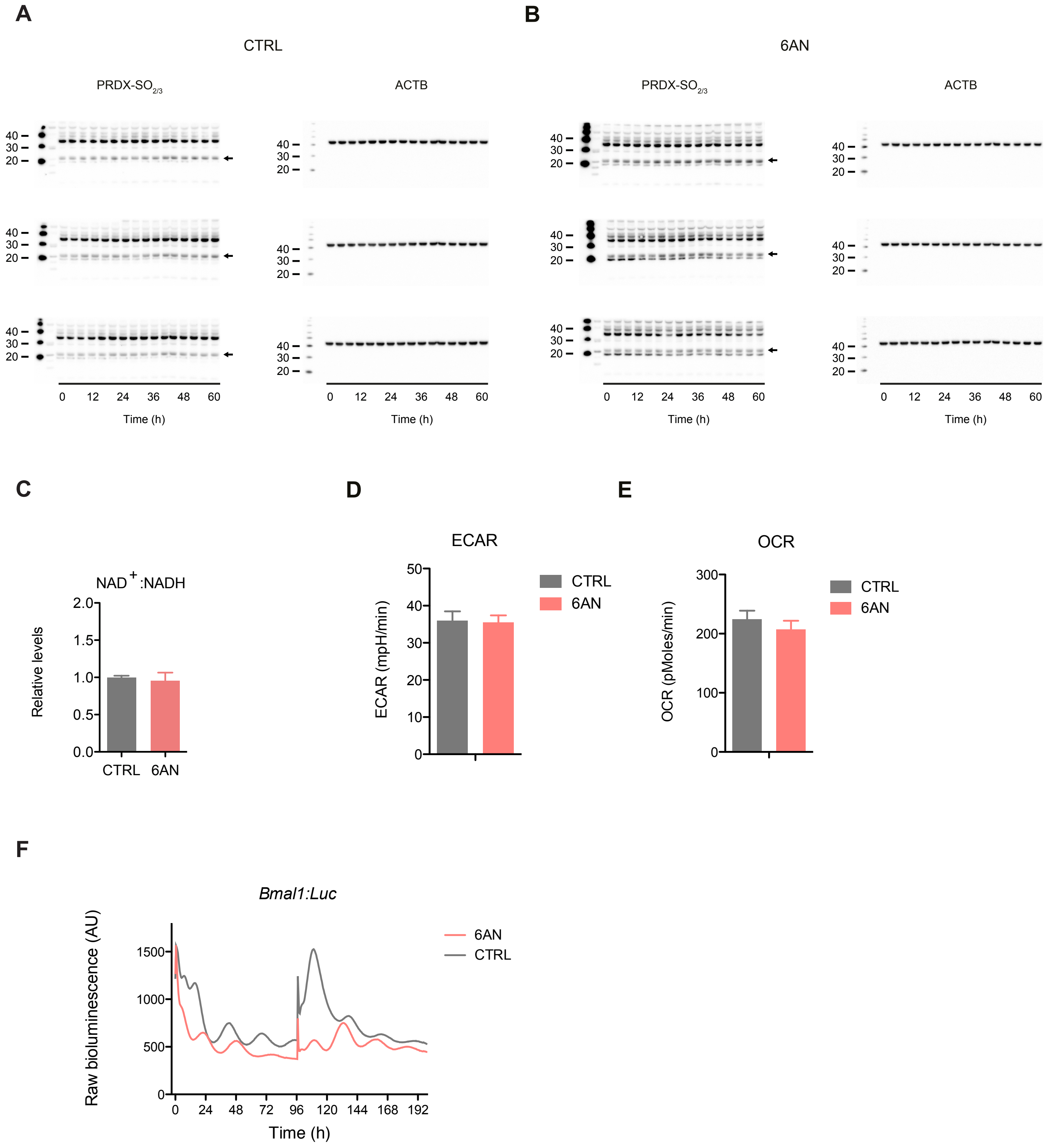
A
B
CTRL
6AN
PRDX-SO2/3
ACTB
40
30
20
0
12
24
36
48
60
Time (h)
C
NAD+:NADH
Relative levels
2.0
1.5
1.0
0.5
0.0
CTRL
6AN
D
ECAR
ECAR (mpH/min)
50
40
30
20
10
0
CTRL
6AN
E
OCR
OCR (pMoles/min)
300
200
100
0
CTRL
6AN
F
Bmal1:Luc
Raw bioluminescence (AU)
1500
1000
500
0
0
24
48
72
96
120
144
168
192
Time (h)
6AN
CTRL

Figure S2

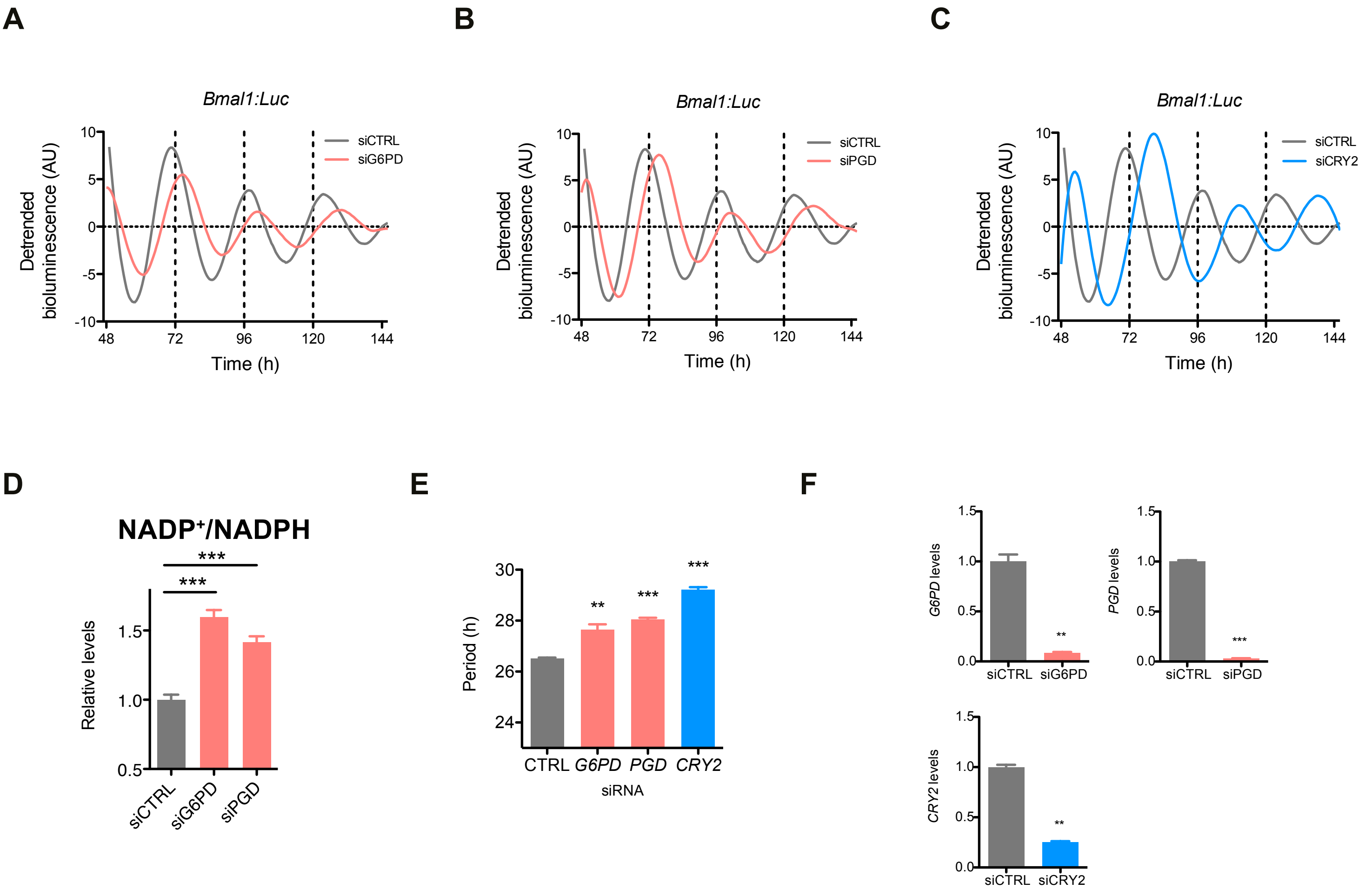
A
B
C
Bmal1:Luc
siCTRL
siG6PD
siPGD
siCRY2
Detrended bioluminescence (AU)
Time (h)
D
NADP⁺/NADPH
Relative levels
siCTRL
siG6PD
siPGD
E
Period (h)
CTRL
G6PD
PGD
CRY2
siRNA
F
G6PD levels
PGD levels
CRY2 levels

Figure S3

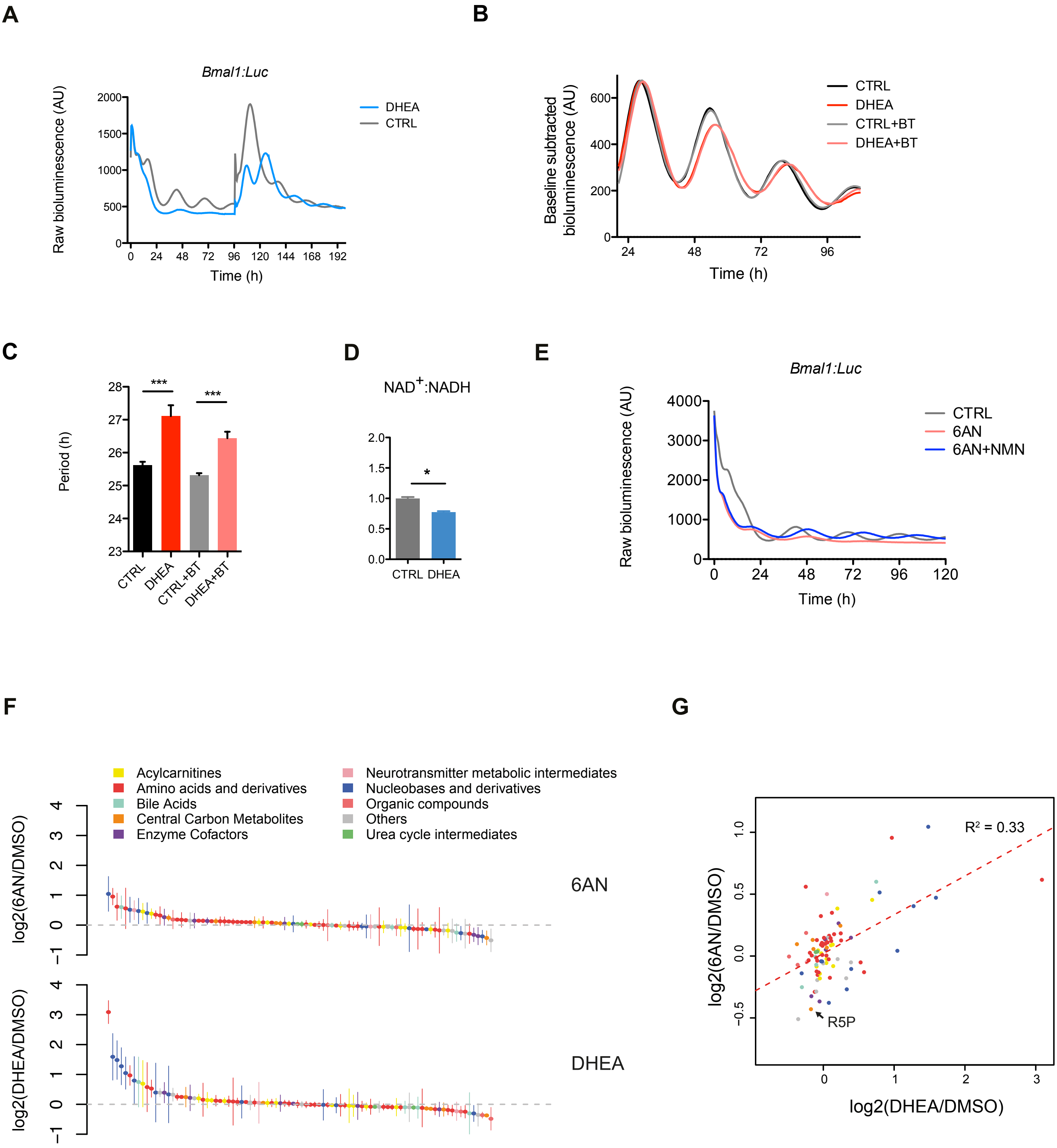

A
Bmal1:Luc
Raw bioluminescence (AU)
2000
1500
1000
500
0
0 24 48 72 96 120 144 168 192
Time (h)
DHEA
CTRL
B
Baseline subtracted bioluminescence (AU)
600
400
200
0
24 48 72 96
Time (h)
CTRL
DHEA
CTRL+BT
DHEA+BT
C
Period (h)
28
27
26
25
24
23
***
***
CTRL
DHEA
CTRL+BT
DHEA+BT
D
NAD+:NADH
2.0
1.5
1.0
0.5
0.0
*
CTRL
DHEA
E
Bmal1:Luc
Raw bioluminescence (AU)
4000
3000
2000
1000
0
0 24 48 72 96 120
Time (h)
CTRL
6AN
6AN+NMN
F
Acylcarnitines
Amino acids and derivatives
Bile Acids
Central Carbon Metabolites
Enzyme Cofactors
Neurotransmitter metabolic intermediates
Nucleobases and derivatives
Organic compounds
Others
Urea cycle intermediates
log2(6AN/DMSO)
4 3 2 1 0 −1
6AN
log2(DHEA/DMSO)
4 3 2 1 0 −1
DHEA
G
R² = 0.33
log2(6AN/DMSO)
1.0
0.5
0.0
−0.5
R5P
0 1 2 3
log2(DHEA/DMSO)

Figure S4

A

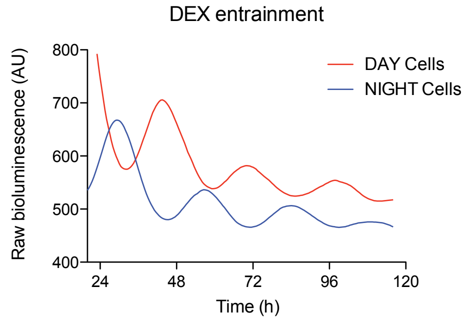

B

	RAIN (p<0.01)	Fisher Test (p<0.05)	Arser (p<0.01)	ALL
**CTRL**	414	820	458	147
**6AN**	453	875	483	169

C

CTRL

Panther GO-Slim Biological Process	Fold Enrichment	Corrected P-value (Bonferri)
transcription from RNA polymerase II promoter (GO:0006366)	1.68	3.67E-02
nucleobase-containing compound metabolic process (GO:0006139)	1.53	2.28E-03
primary metabolic process (GO:0044238)	1.3	1.00E-02
metabolic process (GO:0008152)	1.29	1.39E-03

6AN

Panther GO Biological Process	Fold Enrichment	Corrected P-value (Bonferri)
circadian rhythm (GO:0007623)	5.17	3.09E-03
rhythmic process (GO:0048511)	3.3	3.88E-02
cellular macromolecule metabolic process (GO:0044260)	1.34	2.45E-03
regulation of metabolic process (GO:0019222)	1.33	2.40E-02
macromolecule metabolic process (GO:0043170)	1.29	1.61E-02

D

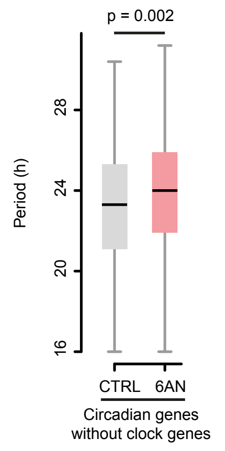

E

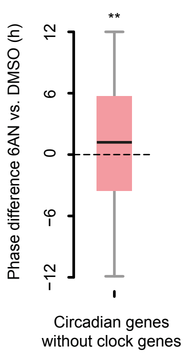

F

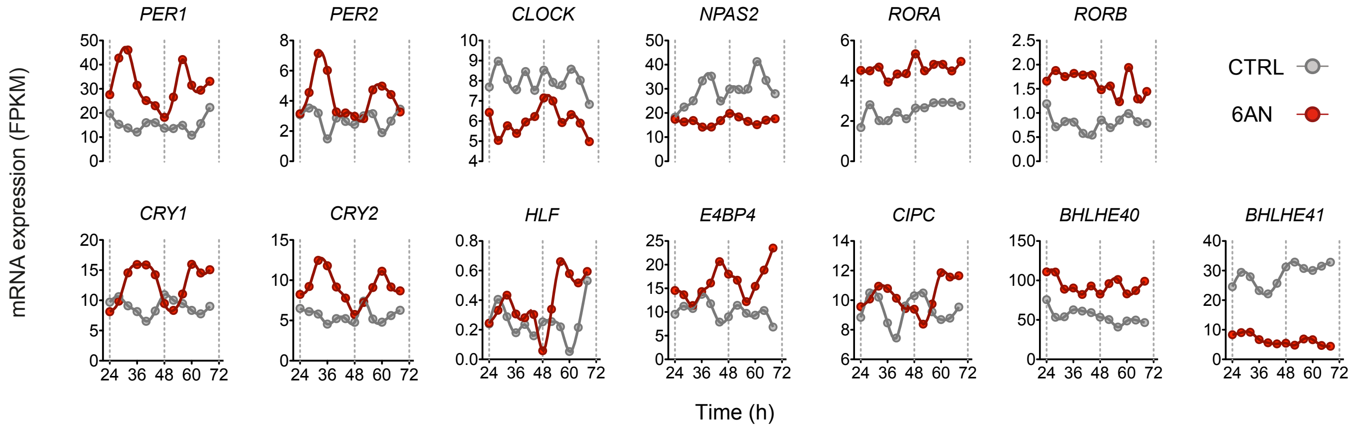

Figure S5

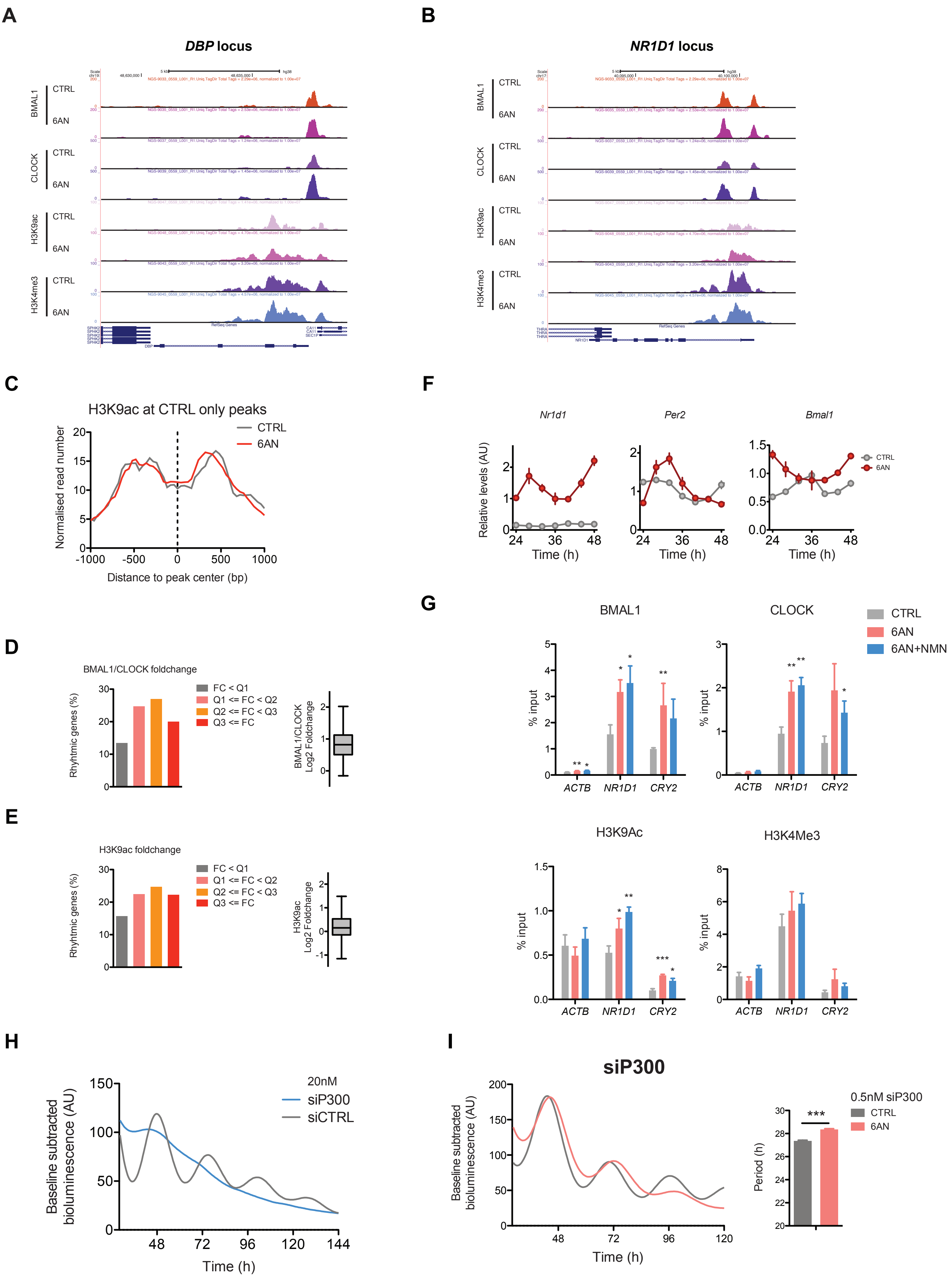

A
DBP locus
BMAL1
CLOCK
H3K9ac
H3K4me3
CTRL
6AN
B
NR1D1 locus
C
H3K9ac at CTRL only peaks
CTRL
6AN
Normalised read number
Distance to peak center (bp)
D
BMAL1/CLOCK foldchange
Rhyhtmic genes (%)
FC < Q1
Q1 <= FC < Q2
Q2 <= FC < Q3
Q3 <= FC
BMAL1/CLOCK Log2 Foldchange
E
H3K9ac foldchange
H3K9ac Log2 Foldchange
F
Nr1d1
Per2
Bmal1
Relative levels (AU)
Time (h)
G
BMAL1
CLOCK
H3K9Ac
H3K4Me3
CTRL
6AN
6AN+NMN
% input
ACTB
NR1D1
CRY2
H
20nM
siP300
siCTRL
Baseline subtracted bioluminescence (AU)
Time (h)
I
siP300
0.5nM siP300
CTRL
6AN
Period (h)
Baseline subtracted bioluminescence (AU)
Time (h)

Figure S6

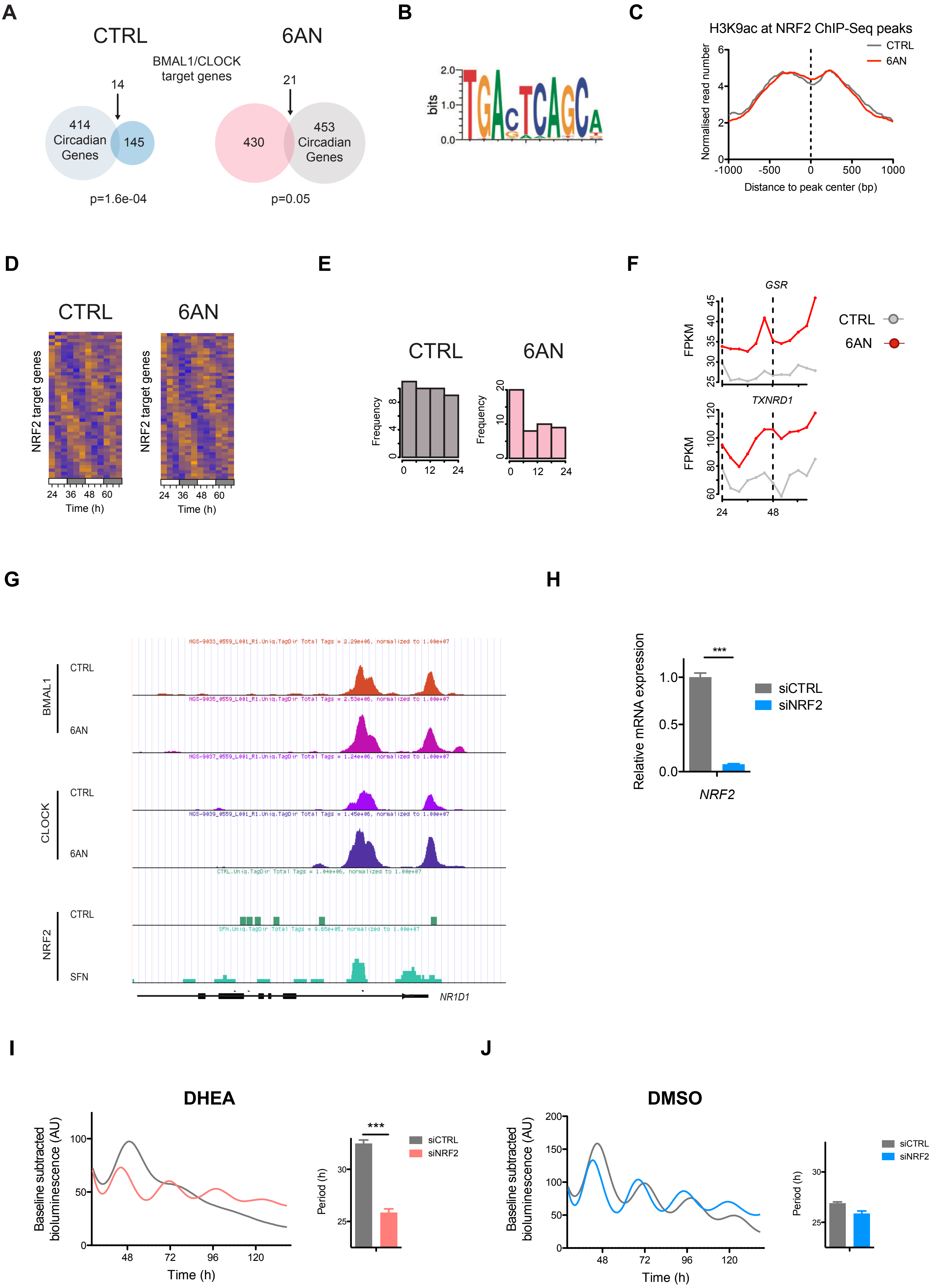
A
CTRL
6AN
BMAL1/CLOCK
target genes
14
414
Circadian
Genes
145
p=1.6e-04
21
430
453
Circadian
Genes
p=0.05
B
bits
2.0
1.0
0.0
C
H3K9ac at NRF2 ChIP-Seq peaks
CTRL
6AN
Normalised read number
Distance to peak center (bp)
D
CTRL
6AN
NRF2 target genes
24 36 48 60
Time (h)
E
CTRL
6AN
Frequency
0 12 24
F
GSR
TXNRD1
FPKM
CTRL
6AN
24
48
G
BMAL1
CLOCK
NRF2
CTRL
6AN
SFN
NR1D1
H
Relative mRNA expression
***
siCTRL
siNRF2
NRF2
I
DHEA
Baseline subtracted
bioluminescence (AU)
Time (h)
Period (h)
***
siCTRL
siNRF2
J
DMSO
Baseline subtracted
bioluminescence (AU)
Time (h)
Period (h)
siCTRL
siNRF2

Figure S7

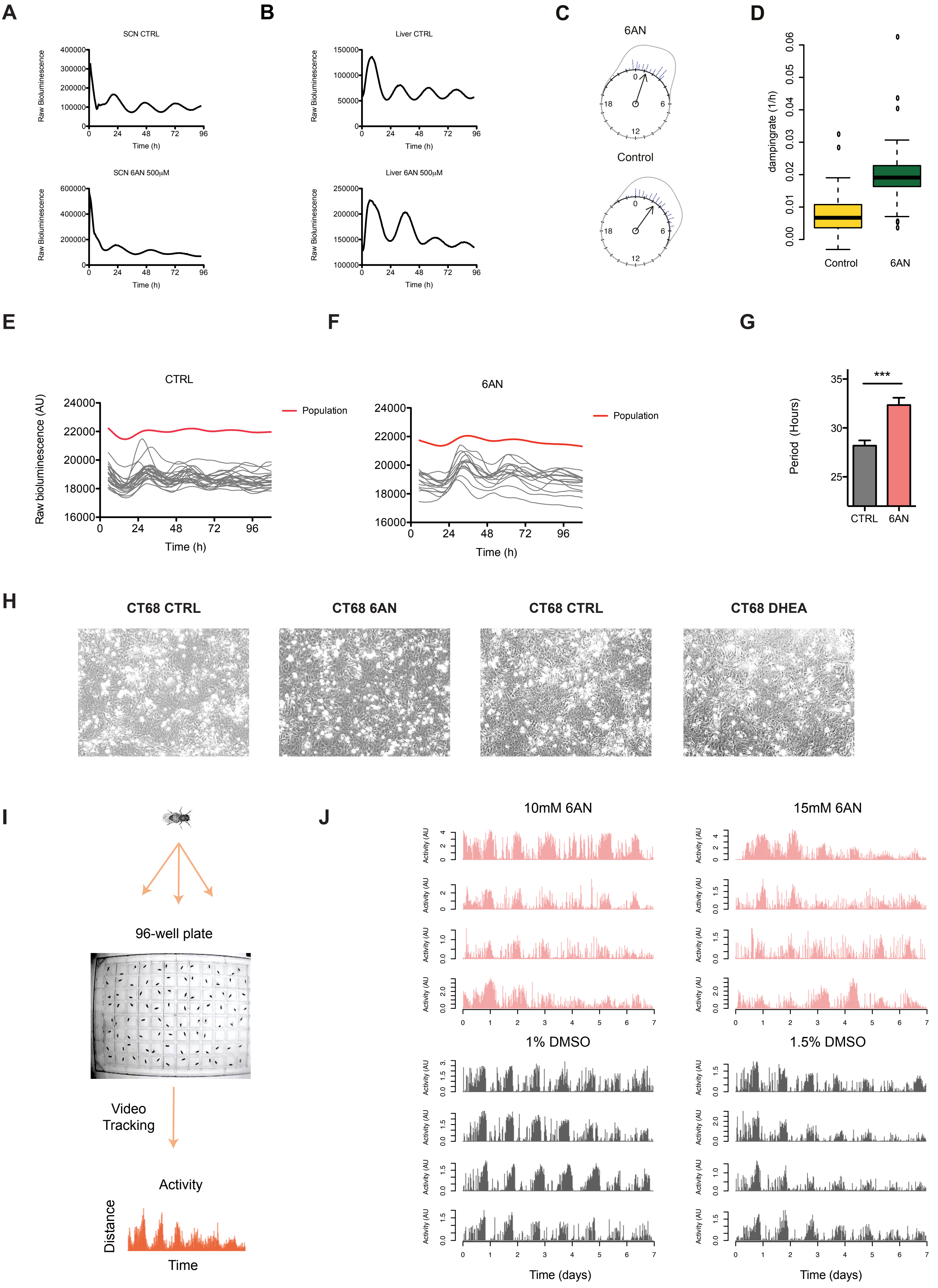

A
SCN CTRL
Raw Bioluminescence
Time (h)
SCN 6AN 500µM
B
Liver CTRL
Liver 6AN 500µM
C
6AN
Control
D
dampingrate (1/h)
Control
6AN
E
CTRL
Raw bioluminescence (AU)
Population
Time (h)
F
6AN
Population
Time (h)
G
Period (Hours)
***
CTRL
6AN
H
CT68 CTRL
CT68 6AN
CT68 CTRL
CT68 DHEA
I
96-well plate
Video Tracking
Activity
Distance
Time
J
10mM 6AN
15mM 6AN
1% DMSO
1.5% DMSO
Time (days)
Time (days)

SUPPLEMENTAL FIGURE LEGENDS

Figure S1. Characterisation of 6AN, related to Figure 1.

(A-B) Immunoblots showing overoxidised peroxiredoxin (PRDX-$SO_{2/3}$) monomers with loading controls (β-actin, ACTB) for *Bmal1*:*luc* U2OS cells treated with 5mM 6AN vs. control (DMSO). Molecular weights (kDa) shown on left side of blots. The band specific to the PRDX-$SO_{2/3}$ monomer is shown with an arrow on the right side of blots. The smile artefact observed between two wells in each blot is an artefact occurring during transfer of NuPAGE Novex Bis-Tris Midi-Gels onto nitrocellulose membranes.

(C) NAD^+:NADH ratio of cells treated with 6AN (mean ± SEM, n=3-4; two-tailed Student's t-test).

(D-E) Measurement of the extracellular acidification rate (ECAR) (C) and oxygen consumption rate (OCR) (D) in *Bmal1*:*luc* U2OS cells shows that acute treatment with 6AN does not affect glycolysis or oxidative phosphorylation (mean ± SEM, n=6-8; two-tailed Student's t-test; not significant).

(F) Raw bioluminescence recordings of *Bmal1*:*luc* U2OS cells treated with 5mM 6AN vs. control (DMSO), followed by wash off after 96h. (mean values shown, n=3-6).

Figure S2. siRNA knockdown of PPP enzymes, related to Figure 1.

(A-C) Bioluminescence recordings of *Bmal1*:*luc* U2OS cells transfected with the indicated siRNAs or control (negative control #1) at a final concentration of 50nM (mean, n=8).

(D) $NADP^+$:NADPH ratio of *Bmal1*:*luc* U2OS cells transfected with the indicated siRNAs (mean ± SEM, n=3-4; two-tailed Student's *t*-test; ; *** $P < 0.001$).

(E) Quantifications of circadian period length of bioluminescence traces from (F-H) (mean ± SEM, n = 8; two-tailed Student's *t*-test; *** $P < 0.001$, ** $P < 0.01$).

(F) qPCR validation of siRNA-mediated knock down efficiency in *Bmal1*:*luc* U2OS. The relative levels of each mRNA were calculated by the $2^{-\Delta\Delta Ct}$ method and normalized to the corresponding *ACTB* mRNA levels (mean ± SEM, n=3; two-tailed Student's *t*-test; *** $P < 0.001$, ** $P < 0.01$).

Figure S3. Characterisation of DHEA and metabolomics analyses, related to Figure 2.

(A) Raw bioluminescence traces for *Bmal1*:*luc* U2OS cells treated with 50 µM dehydroepiandrosterone (DHEA) vs. control (DMSO), followed by wash off after 96h (mean values shown, n=3-6).

(B) Bioluminescence recordings of *Per2*:*luc* U2OS cells treated with the indicated drugs. Treatment with 200nM Bicalutamide (BT), an anti-androgen drug, does not suppress the effect of DHEA on circadian oscillations (mean values shown, n=8).

(C) Quantifications of the period length from (B) (mean ± SEM, n=8; two-tailed Student's *t*-test; *** $P < 0.001$).

(D) NAD^+:NADH ratio of cells treated with DHEA (mean ± SEM, n=3-4; two-tailed Student's *t*-test; * $P < 0.05$).

(E) Raw bioluminescence traces for *Bmal1*:*luc* U2OS cells treated with 6AN, or 6AN and NMN, vs. control (DMSO) (mean values shown, n=8).

(F) Metabolomics profiling in *Bmal1*:*luc* U2OS treated with 5mM 6AN vs. control (DMSO) (top) or 50 μM DHEA vs. control (bottom) (mean ± SEM, n=3).

(G) The log fold change exhibits a significant correlation between the 6AN and DHEA conditions (linear regression in log space, $R^2 = 0.33$, $p<1x10^{-4}$). R5P, Ribose 5-phosphate.

Figure S4. RNA-Seq in *Bmal1:Luc* U2OS cells, related to Figure 3.

(A) For mRNA time course experiments, *Bmal1*:*luc* U2OS cells were treated with 100 nM dexamethasone at 12 h intervals to differentially entrain the cells (i.e. a population of Day vs. Night cells). Bioluminescence recordings confirm that the two sets of cells exhibit antiphasic oscillation of the reporter gene (median, n=24).

(B) Comparison of different algorithms to detect circadian transcripts in the control (DMSO) and 6AN RNA-Seq datasets. For each algorithm, the number of rhythmic genes found at the given threshold is given. In the last column, the number of genes common to the three methods is shown.

(C) Gene ontology (GO) analysis of 414 and 453 circadian genes in the control (DMSO) and 6AN conditions respectively. Enrichment of GO terms for biological processes was performed with PANTHER database.

(D) Distribution of period length for 6AN and control mRNA profiles for circadian transcripts (RAIN algorithm, p≤0.01) excluding clock gene transcripts (Table S4). (Wilcoxon rank sum test, with p-values as shown).

(E) Distribution of phase differences between 6AN and control mRNA profiles for circadian transcripts (RAIN algorithm, p≤0.01) excluding clock gene transcripts (Kuiper's one-sample test of uniformity, * $P < 0.01$).

(F) Profiles of mRNA accumulations for indicated clock genes. FPKM, Fragments Per Kilobase of transcript per Million.

Figure S5. BMAL1/CLOCK ChIP analyses and *P300* siRNA, related to Figure 4.

(A-B) ChIP-Seq data at the *DBP* (A) and *NR1D1* (B) loci (visualized in the UCSC Genome Browser) showing BMAL1, CLOCK, H3K9ac and H3K4me3 tracks with control and 6AN conditions.

(C) Genomic profiles of H3K9ac densities around BMAL1/CLOCK peaks specific to the control condition.

(D-E) Fraction of circadian genes depending of the fold change in BMAL1/CLOCK binding (D) and H3K9ac levels (E). Genes near BMAL1/CLOCK 6AN peaks were stratified in four groups using quartiles Q1, Q2 and Q3 (respectively p=0.25, 0.5 and 0.75) using the distribution of fold change for BMAL1/CLOCK 6AN vs. CTRL (D) and H3K9ac 6AN vs. CTRL (E). For each group, the fraction of genes with $p<0.05$ using the RAIN algorithm is shown. Boxplot of fold changes for BMAL1/CLOCK and H3K9ac are shown (right).

(F) Time course of mRNA expression determined by RT-qPCR in *SIRT1*$^{-/-}$ MEFs incubated with 5mM 6AN (red) or control (DMSO, grey) (mean ± SEM, n=3). Cells were synchronized with a dexamethasone shock and total RNA was collected at the indicated time points.

(G) BMAL1, CLOCK, H3K9ac and H3K4me3 ChIP-qPCR in *Bmal1*:*luc* U2OS cells following 24 h control (DMSO), 6AN or 6AN+NMN treatment are shown. (mean ± SEM, n=3; two-tailed Student's *t*-test; *** $P < 0.001$, ** $P < 0.01$, * $P < 0.05$).

(H) Bioluminescence recordings of *Bmal1*:*luc* U2OS cells transfected with *P300* siRNA at a final concentration of 20nM. As a control, Non-Targeting siRNA #1 was used at the same concentration (mean, n=8-16).

(I) Bioluminescence recordings of *Bmal1*:*luc* U2OS cells transfected with *P300* siRNA at a final concentration of 0.5nM treated with 6AN or control (DMSO) (left; mean, n=8). Quantifications of circadian period length of bioluminescence traces (right; mean ± SEM, n = 8; two-tailed Student's *t*-test; *** $P < 0.001$).

Figure S6. NRF2 targets exhibit circadian gene expression, related to Figure 5.

(A) Venn diagrams showing the overlap between circadian genes and genes bound by BMAL1/CLOCK in the control and 6AN conditions (Fisher test on contingency table, with p-values as shown).

(B) NRF2-binding motif determined by ChIP-Seq analyses from (Chorley et al., 2012).

(C) Genomic profiles of H3K9ac densities around the 849 NRF2 ChIP-Seq peaks from (Chorley et al., 2012).

(D) Heat map representation of the temporal accumulation of mRNA for NRF2 target genes detected as circadian in the 6AN and control conditions.

(E) Phase histogram of rhythmic transcripts shown in (D).

(F) Profiles of mRNA accumulations for *Glutathione Reductase* (*GSR*) and *Thioredoxin Reductase 1* (*TXNRD1*). FPKM, Fragments Per Kilobase of transcript per Million.

(G) ChIP-Seq data at the *NR1D1* locus (visualized in the UCSC Genome Browser) showing two peaks located at the promoter and in the first intron. Both sites are bound by BMAL1/CLOCK and NRF2. NRF2 data are from (Chorley et al., 2012).

(H) qPCR validation of *NRF2* siRNA-mediated knock down efficiency in *Bmal1*:*luc* U2OS. The relative levels of *NRF2* were calculated by the $2^{-\Delta\Delta Ct}$ method and normalized to the corresponding *ACTB* mRNA levels (mean ± SEM, n=3; two-tailed Student's *t*-test).

(I-J) Bioluminescence recordings of *Bmal1*:*luc* U2OS cells transfected with 20nM *NRF2* siRNA or control (Non-Targeting siRNA #1) combined with DHEA treatment at 25 µM (I) or with control (DMSO) (J) (left; mean, n=8). Quantifications of circadian period length of bioluminescence traces (right; mean ± SEM, n = 8; two-tailed Student's *t*-test; *** $P < 0.001$).

Figure S7. Bioluminescence data in mouse tissues and U2OS cells, and fly behavioural recording system, related to Figure 6 and Figure 7.

(A-B) Raw bioluminescence traces of suprachiasmatic nuclei (SCN) (A) and liver slices (B) from *mPer2*^{Luciferase} (*mPer2*^{Luc}) mice treated with the indicated concentration of 6AN or control (DMSO).

(C) Rayleigh plot showing the distribution of circadian phases at the beginning of the traces shown in Figure 6E.

(D) Boxplot showing the difference in estimated damping rate between 6AN and control cells (two-tailed Student's *t*-test, P = $1x10^{-16}$).

(E-F) Single-cell bioluminescence traces of *Per2*:*luc* U2OS cells treated with control (DMSO, (E)) or 6AN (F). The average of the whole population is shown in red.

(G) Quantifications of the period length from (E-F) (mean ± SEM, n>10; two-tailed Student's *t*-test; *** $P < 0.001$).

(H) Brightfield images of *Bmal1*:*luc* U2OS cells treated with 5mM 6AN or control (0.5% DMSO), and 50µM DHEA or control (0.5% EtOH) for 68h.

(I) Schematic showing the workflow for high-throughput fly behavioural recordings. Individual flies were placed into wells of a 96-well plate and, using a custom made infra-red video recording system, the locomotor activity of individual 4-7 day old flies was recorded in constant darkness (DD). The videos were processed to quantify the locomotor activity of the flies and the distance travelled per minute was the measure of the flies' locomotor activity.

(J) For comparison with Figure 7C, representative activity plots of individual flies following treatment with 6AN or control.

SUPPLEMENTAL TABLES

Table S1. List of siRNAs used in this study. Related to Figure S2, Figure S5, Figure 5 and Figure S6.

Gene Symbol	Catalog Number	Supplier
Negative Control No. 1	4390843	Life Technologies
Non-Targeting siRNA No. 1	D-001810-01	Dharmacon
CRY2	L-014151-01	Dharmacon
G6PD	L-008181-02	Dharmacon
PGD	L-008371-00	Dharmacon
NRF2	L-003755-00	Dharmacon
EP300	L-003486-00	Dharmacon

Table S2. List of primers used in this study. Related to Figure 3 and 5, and Figure S2, S5, and S6.

RT-qPCR

Gene	Species	Detection	Assay ID	Supplier
ACTB	Human	Taqman	Hs01060665_g1	Life Technologies
CRY2	Human	Taqman	Hs00323654_m1	Life Technologies
G6PD	Human	Taqman	Hs00166169_m1	Life Technologies
PGD	Human	Taqman	Hs00427230_m1	Life Technologies
BMAL1	Human	Taqman	Hs00154147_m1	Life Technologies
DBP	Human	Taqman	Hs00609747_m1	Life Technologies
NR1D1	Human	Taqman	Hs00253876_m1	Life Technologies
NR1D2	Human	Taqman	Hs00233309_m1	Life Technologies
PER3	Human	Taqman	Hs00213466_m1	Life Technologies
TEF	Human	Taqman	Hs01115720_m1	Life Technologies
NRF2	Human	Taqman	Hs00975961_g1	Life Technologies
ACTB	Mouse	Taqman	Mm00607939_s1	Life Technologies
NR1D1	Mouse	Taqman	Mm00520708_m1	Life Technologies
ARNTL	Mouse	Taqman	Mm00500226_m1	Life Technologies
PER2	Mouse	Taqman	Mm00478113_m1	Life Technologies

ChIP-qPCR

Gene	Species	Type	Sequence (5' -> 3')	Detection
NR1D1	Human	Forward	CTACGTTCCCTCGGCAGTAA	SYBR Green
NR1D1	Human	Reverse	TCACATGGTACCTGCTCCAG	SYBR Green
NR1D1 1st intron	Human	Forward	CGCTTCCCGTCAATCGAGA	SYBR Green
NR1D1 1st intron	Human	Reverse	GTTGTCCTGGCCCTGCTATC	SYBR Green
PER1	Human	Forward	AGACCTCTCAGCCTATGAGAAAGC	SYBR Green
PER1	Human	Reverse	CCCGACCTGCCAAGATTG	SYBR Green
CRY2	Human	Forward	TGGGTAAGAGATCCGCTGTC	SYBR Green
CRY2	Human	Reverse	CCCTCACGTTCCTACCATGT	SYBR Green
HMOX1	Human	Forward	ATTTCCTCATCCCCTCGTGC	SYBR Green
HMOX1	Human	Reverse	AGCAAAATCCGCCTTCCCTT	SYBR Green
DBP	Human	Forward	AAACACGGACCAATCGTCTC	SYBR Green
DBP	Human	Reverse	GAAAGGCAAGGCAACTTCAC	SYBR Green
ACTB	Human	Forward	CACCGTCCGTTGTATGTCTG	SYBR Green
ACTB	Human	Reverse	GCTTTGAGTTCCTAGCACCG	SYBR Green

Table S3. Metabolomics data. Related to Figure S3.

Metabolite	DMSO_A	DMSO_B	DMSO_C	6AN_A	6AN_B	6AN_C	DHEA_A	DHEA_B	DHEA_C
UDP-Glucose	6.10E+02	6.04E+02	5.72E+02	6.44E+02	6.41E+02	5.88E+02	6.10E+02	6.22E+02	6.15E+02
Choline	5.68E+01	7.03E+01	4.17E+01	6.46E+01	2.67E+01	3.92E+01	3.05E+01	4.63E+01	6.69E+01
Taurine	1.81E+02	1.71E+02	1.35E+02	1.94E+02	1.45E+02	1.26E+02	1.37E+02	1.41E+02	1.64E+02
Glycine	1.22E+02	1.06E+02	8.59E+01	1.16E+02	8.66E+01	9.89E+01	1.03E+02	9.47E+01	9.33E+01
L-Glutamic Acid	2.75E+02	2.56E+02	2.29E+02	2.82E+02	2.40E+02	2.31E+02	2.29E+02	2.31E+02	2.45E+02
Myoinositol	6.64E+01	6.88E+01	6.22E+01	6.88E+01	6.86E+01	7.77E+01	5.71E+01	6.04E+01	6.01E+01
Alanine	7.89E+01	7.95E+01	6.58E+01	9.43E+01	8.26E+01	7.42E+01	7.28E+01	8.01E+01	8.20E+01
Creatine	1.24E+02	1.28E+02	1.01E+02	1.43E+02	1.32E+02	1.04E+02	1.14E+02	1.19E+02	1.13E+02
Aspartate	5.55E+01	5.27E+01	2.61E+01	5.90E+01	2.64E+01	2.33E+01	2.48E+01	2.72E+01	4.69E+01
Proline	6.21E+01	6.48E+01	6.84E+01	6.81E+01	7.78E+01	6.95E+01	7.39E+01	7.78E+01	5.67E+01
Homocysteine	5.25E+01	4.80E+01	4.34E+01	3.52E+01	4.45E+01	4.95E+01	3.89E+01	5.11E+01	4.61E+01
AMP	8.24E+00	8.03E+00	2.06E+01	9.53E+00	1.00E+01	1.41E+01	1.41E+01	2.82E+01	1.99E+01
Cysteine	2.25E+01	2.30E+01	2.17E+01	2.39E+01	2.23E+01	2.14E+01	2.11E+01	2.23E+01	2.31E+01
Spermidine	3.83E+01	4.12E+01	4.11E+01	3.90E+01	3.66E+01	3.99E+01	4.12E+01	4.36E+01	3.55E+01
Valine	1.82E+01	1.91E+01	1.75E+01	1.97E+01	2.02E+01	1.82E+01	1.84E+01	2.01E+01	1.59E+01
Acetoacetic acid	5.44E+01	7.71E+01	5.81E+01	7.27E+01	3.92E+01	4.52E+01	5.95E+01	7.88E+01	4.15E+01
Serine	2.03E+01	2.19E+01	1.55E+01	2.12E+01	1.86E+01	1.73E+01	1.70E+01	1.57E+01	1.74E+01
Threonine	2.25E+01	2.60E+01	1.82E+01	2.22E+01	2.02E+01	2.15E+01	2.39E+01	2.59E+01	2.01E+01
Leucine	1.39E+01	1.55E+01	1.41E+01	1.59E+01	1.66E+01	1.48E+01	1.51E+01	1.76E+01	1.43E+01
Ornithine	8.70E+00	9.67E+00	8.76E+00	9.19E+00	9.65E+00	8.94E+00	8.51E+00	9.02E+00	8.06E+00
IMP	2.02E+00	2.48E+00	7.38E+00	3.32E+00	3.84E+00	5.72E+00	4.86E+00	9.78E+00	6.71E+00
deoxycytidine	7.26E+00	7.46E+00	5.33E+00	7.88E+00	6.27E+00	5.26E+00	5.75E+00	5.91E+00	7.21E+00
Glyceraldehyde	1.36E+01	1.36E+01	9.92E+00	1.14E+01	1.43E+01	1.31E+01	1.05E+01	1.03E+01	7.80E+00
Arginine	8.44E+00	8.58E+00	8.57E+00	9.35E+00	9.15E+00	8.91E+00	9.13E+00	9.45E+00	7.52E+00
Histidine	1.11E+01	1.16E+01	1.12E+01	1.13E+01	9.38E+00	1.03E+01	1.06E+01	1.18E+01	9.32E+00
Lysine	8.66E+00	9.84E+00	7.56E+00	9.97E+00	1.13E+01	8.38E+00	1.00E+01	1.15E+01	9.35E+00
S-5-Adenosyl-L-Methionine	6.08E+00	1.07E+01	6.29E+00	5.22E+00	6.67E+00	1.85E+01	5.71E+00	6.56E+00	6.12E+00
Betaine	1.55E+00	1.18E+00	1.69E+00	1.45E+00	1.81E+00	1.43E+00	1.38E+00	1.67E+00	1.39E+00
Isoleucine	5.63E+00	5.13E+00	5.48E+00	5.80E+00	5.96E+00	5.61E+00	5.51E+00	5.79E+00	4.77E+00
D-Ribose 5-phosphate	4.16E+00	3.79E+00	4.91E+00	3.38E+00	3.38E+00	2.57E+00	2.92E+00	4.45E+00	3.83E+00
Phenylalanine	5.86E+00	5.82E+00	5.82E+00	6.27E+00	6.60E+00	5.96E+00	5.81E+00	6.30E+00	5.16E+00
Sorbitol	1.80E+00	1.58E+00	1.56E+00	1.67E+00	1.70E+00	1.74E+00	1.63E+00	1.55E+00	1.31E+00
Hydroxyproline	4.13E+00	4.44E+00	3.46E+00	4.53E+00	3.59E+00	3.54E+00	3.21E+00	3.22E+00	3.78E+00
NAD	1.53E+01	1.39E+01	1.34E+01	1.56E+01	1.77E+01	1.35E+01	1.45E+01	1.44E+01	1.43E+01
Tyrosine	4.31E+00	4.42E+00	4.58E+00	4.77E+00	5.21E+00	4.74E+00	4.89E+00	4.61E+00	3.86E+00
Glycocholic Acid	2.64E+00	2.28E+00	1.63E+00	2.90E+00	2.07E+00	1.60E+00	1.65E+00	1.69E+00	2.30E+00
Succinate	8.52E+00	8.69E+00	5.73E+00	1.07E+01	1.12E+01	5.84E+00	6.48E+00	9.54E+00	9.66E+00
Asparagine	3.54E+00	3.77E+00	2.92E+00	3.81E+00	4.24E+00	3.16E+00	3.38E+00	3.50E+00	3.45E+00
Citrulline	3.13E+00	3.88E+00	3.30E+00	3.52E+00	3.47E+00	3.45E+00	2.80E+00	3.40E+00	3.36E+00
Glutamine	4.77E+00	6.13E+00	5.00E+00	5.57E+00	5.21E+00	5.19E+00	4.23E+00	4.31E+00	5.21E+00
L-Methionine	2.23E+00	2.47E+00	2.35E+00	2.64E+00	2.75E+00	2.20E+00	2.49E+00	2.83E+00	2.02E+00
Carnitine	1.51E+00	1.36E+00	1.12E+00	1.49E+00	1.56E+00	1.08E+00	1.24E+00	1.26E+00	1.24E+00
Aminodipic Acid	1.23E+00	1.94E+00	8.24E-01	1.88E+00	6.76E-01	8.39E-01	2.09E+00	1.80E+00	1.39E+00
Propionylcarnitine	1.13E+00	1.16E+00	7.66E-01	1.24E+00	9.28E-01	7.17E-01	8.10E-01	8.65E-01	1.03E+00
Allantoin	1.11E+00	1.02E+00	1.39E+00	1.03E+00	1.08E+00	1.25E+00	1.73E+00	1.15E+00	1.72E+00
Tryptophan	1.69E+00	1.64E+00	1.77E+00	1.41E+00	2.09E+00	1.60E+00	1.84E+00	1.88E+00	1.52E+00
2-deoxyuridine	6.97E-01	3.39E-01	3.30E-01	3.94E-01	3.04E-01	2.80E-01	3.12E-01	3.91E-01	5.18E-01
D-Glucuronic acid	1.51E+00	2.08E+00	1.52E+00	1.66E+00	1.29E+00	1.73E+00	1.10E+00	1.48E+00	1.36E+00
S-Adenosyl-L-Homocysteine	8.59E-01	1.10E+00	8.81E-01	9.61E-01	9.64E-01	1.55E+00	8.60E-01	9.45E-01	9.44E-01
Niacinamide	4.33E+00	5.41E+00	5.10E+00	6.38E+00	5.60E+00	4.16E+00	6.43E+00	9.03E+00	3.97E+00
Pantothenic Acid	1.25E+00	1.03E+00	1.69E+00	1.15E+00	1.73E+00	1.69E+00	1.58E+00	1.67E+00	1.01E+00
Acetylcarnitine	3.12E-01	3.46E-01	6.72E-01	3.37E-01	6.69E-01	7.34E-01	6.86E-01	7.61E-01	2.93E-01
Asymmetric dimethylarginine	8.62E-01	8.59E-01	7.72E-01	1.31E+00	1.01E+00	8.73E-01	7.18E-01	1.05E+00	9.33E-01
Carnosine	3.76E-01	3.84E-01	3.91E-01	3.89E-01	3.80E-01	3.62E-01	4.34E-01	4.77E-01	4.13E-01
Adenosine	5.85E-02	4.76E-02	1.96E-01	8.84E-02	9.17E-02	1.41E-01	1.23E-01	2.86E-01	1.82E-01
Cystathionine	1.59E+00	1.93E+00	1.41E+00	2.06E+00	1.74E+00	1.52E+00	1.49E+00	1.67E+00	1.42E+00
Phosphoethanolamine	4.10E-02	5.84E-02	6.36E-02	3.70E-02	4.16E-02	6.57E-02	6.09E-02	7.94E-02	5.77E-02
Nicotinic Acid	1.45E-01	1.61E-01	1.16E-01	1.54E-01	8.45E-02	8.62E-02	9.02E-02	1.03E-01	1.89E-01
GABA	1.36E-01	1.38E-01	1.94E-01	2.01E-01	2.60E-01	1.72E-01	7.65E-02	2.89E-01	8.87E-02
Creatinine	4.01E-01	2.45E-01	3.63E-01	3.07E-01	3.03E-01	2.54E-01	3.02E-01	3.20E-01	2.83E-01
3-Hydroxyanthranilic acid	1.15E-01	1.08E-01	1.33E-01	1.48E-01	2.61E-01	2.82E-01	1.76E-01	3.16E-01	1.88E-01
Symmetric dimethylarginine	1.25E-01	5.29E-02	2.06E-01	5.71E-02	9.73E-02	9.14E-02	1.49E-01	1.52E-01	8.21E-02
Guanidinoacetic Acid	7.38E-02	9.91E-02	1.86E-01	1.22E-01	7.37E-02	4.90E-02	1.24E-01	1.23E-01	5.27E-02
L-Kynurenine	1.44E-01	1.58E-01	9.72E-02	1.80E-01	1.39E-01	1.11E-01	1.51E-01	1.80E-01	1.34E-01
Gamma-Glutamylcysteine	8.86E-02	1.67E-01	9.94E-02	8.83E-02	6.71E-02	7.06E-02	1.09E-01	9.28E-02	5.59E-02
Palmitoyl Carnitine	1.09E-01	1.17E-01	7.53E-02	1.17E-01	7.10E-02	8.71E-02	8.84E-02	1.16E-01	1.13E-01
Isovalerylcarnitine	1.01E-01	9.91E-02	4.84E-02	1.05E-01	6.06E-02	4.80E-02	5.21E-02	5.86E-02	8.67E-02
5-Hydroxyindole-3-acetic acid	7.59E-02	9.20E-02	6.14E-02	1.14E-01	7.49E-02	3.57E-02	4.62E-02	6.51E-02	7.59E-02
Isobutyrylcarnitine	2.13E-01	1.91E-01	2.09E-01	2.32E-01	3.06E-01	2.55E-01	2.30E-01	2.63E-01	2.06E-01
Hypoxanthine	1.73E-02	1.09E-02	7.69E-03	1.43E-02	3.52E-02	1.65E-02	1.83E-02	5.48E-02	1.78E-02
Stearoyl Carnitine	6.85E-02	7.28E-02	7.07E-02	7.86E-02	6.51E-02	8.17E-02	7.43E-02	8.77E-02	7.07E-02
Xanthosine	8.46E-02	6.71E-02	8.07E-02	7.20E-02	6.42E-02	7.40E-02	4.69E-02	7.08E-02	6.60E-02
Folic Acid	6.15E-02	4.66E-02	5.67E-02	6.37E-02	3.56E-02	3.37E-02	3.81E-02	4.66E-02	5.94E-02
Guanosine	2.78E-02	3.84E-02	1.39E-02	1.65E-02	3.36E-02	1.41E-02	1.35E-02	1.35E-02	4.07E-02
DimethylGlycine	3.38E-02	2.69E-02	3.99E-02	2.74E-02	3.48E-02	4.35E-02	3.33E-02	3.69E-02	2.30E-02
Taurocholic Acid	2.02E-02	2.09E-02	4.53E-02	2.11E-02	4.99E-02	5.11E-02	4.79E-02	4.74E-02	1.84E-02
Arachidyl Carnitine	2.33E-02	2.25E-02	2.29E-02	2.33E-02	2.33E-02	2.30E-02	2.29E-02	2.24E-02	2.19E-02
Cytidine	1.00E-02	1.55E-02	2.99E-02	1.27E-02	3.43E-02	2.40E-02	2.82E-02	3.01E-02	1.36E-02
Hippuric acid	1.02E-02	6.80E-03	1.27E-02	4.52E-03	1.04E-02	1.30E-02	9.09E-03	2.70E-03	1.08E-02
Taurochenodeoxycholic Acid	1.74E-02	1.17E-02	6.69E-03	1.43E-02	6.53E-03	7.61E-03	9.76E-03	3.52E-03	1.05E-02
cGMP	7.86E-03	7.70E-03	2.89E-02	1.28E-02	6.93E-03	7.44E-03	1.48E-02	1.40E-02	6.88E-03
Myristoyl Carnitine	1.72E-02	1.75E-02	1.32E-02	1.80E-02	1.25E-02	1.48E-02	1.57E-02	1.60E-02	1.67E-02
2-Aminoisobutyric acid	8.87E-03	4.51E-03	8.87E-03	7.11E-03	6.48E-03	1.02E-02	8.06E-03	8.66E-03	4.48E-03
cAMP	1.38E-02	9.87E-03	1.03E-02	2.11E-02	7.24E-03	6.67E-03	6.47E-03	1.60E-02	9.04E-03
Dodecanoyl Carnitine	5.88E-03	5.38E-03	4.85E-03	5.55E-03	4.59E-03	5.59E-03	5.45E-03	5.06E-03	5.96E-03
Hexanoylcarnitine	3.81E-03	3.67E-03	3.94E-03	3.83E-03	4.27E-03	4.00E-03	4.51E-03	4.17E-03	3.61E-03
Pyridoxine	6.21E-03	6.90E-03	5.10E-03	7.59E-03	5.22E-03	5.75E-03	5.88E-03	5.00E-03	7.15E-03
1-methylhistamine	5.90E-04	7.62E-04	1.06E-03	8.85E-04	9.10E-04	7.62E-04	4.43E-04	8.12E-04	7.62E-04
Decanoylcarnitine	1.21E-03	6.34E-04	9.37E-04	9.47E-04	5.27E-04	1.03E-03	8.59E-04	9.47E-04	6.54E-04
Kynurenic Acid	1.95E-04	5.37E-04	1.95E-04	4.88E-04	4.56E-04	2.44E-04	2.03E-03	2.51E-03	2.03E-03

Table S4. List of clock genes. Related to Figure 3 and Figure 4.

Clock genes	Direct BMAL1/CLOCK genes	Indirect BMAL1/CLOCK genes
NR1D1	NR1D1	PER1
NR1D2	NR1D2	PER2
DBP	DBP	PER3
TEF	TEF	CRY1
HLF	HLF	CRY2
PER1	BHLHE40	RORA
PER2	BHLHE41	RORB
PER3		RORC
CRY1		NFIL3
CRY2		
ARNTL		
CLOCK		
NPAS2		
NFIL3		
BHLHE40		
BHLHE41		
RORA		
RORB		
RORC		
CIPC		

SUPPLEMENTAL EXPERIMENTAL PROCEDURES

Cell culture and bioluminescence assays

Bmal1:*luc* U2OS and *Per2*:*luc* U2OS cells were a gift from Dr Andrew Liu, University of Memphis, USA (Liu et al., 2008). U2OS cells were cultured in Dulbecco's modified Eagle medium (DMEM) containing 4.5 g/l glucose (Sigma D6546), 10% (v/v) Newborn Calf Serum (Sigma 12023C), 1X GlutaMAX™, 100 U penicillin/ml and 100 µg/ml streptomycin (Penicillin-Streptomycin Solution, Sigma P0781), 1X MycoZap™ Plus-PR (Lonza) and blasticidin 2 µg/ml. Cells were cultured at 37°C, 5% CO_2 in a standard humidified incubator. For bioluminescence recordings, U2OS cells were grown to confluence and synchronised by changing medium to "Air Medium" (Hastings et al., 2005): DMEM (Sigma D5030) supplemented with 5 g/l glucose (Sigma G8644), 20mM HEPES (Sigma H0887), 100 U penicillin/ml and 100 µg/ml streptomycin, 0.035% NaHCO3 (Sigma S8761), 10% Newborn Calf Serum (Sigma 12023C), 1X GlutaMAX™ (Life Technologies 35050-038), 0.5X B-27® Supplement (Life Technologies 17504-044), 1 mM Luciferin (Biosynth L8220), 1X Non-Essential Amino acids (Sigma M7145), 1X MycoZap™ Plus-PR (Lonza) and blasticidin 2 µg/ml. 6AN was made up as a 1M stock in DMSO solvent by briefly heating at 37°C. DHEA was made up in DMSO or EtOH. Bicalutamide (Sigma B9061) was made up as a 4mM stock in DMSO. Drugs were then added the Air medium to reach a final concentration of 5mM (6AN), 50µM (DHEA) and 200nM (Bicalutamide). β-Nicotinamide mononucleotide (NMN) (Sigma N3501) was directly in dissolved in cell culture media at the indicated concentration.

Bioluminescence assays were performed at 37°C using 12-well and 96-well plates in custom-made bioluminescence recording systems (Cairn Research Ltd, Faversham, UK) composed of a charge-coupled device (CCD) camera (Andor iKon-M 934) mounted on the top of an Eppendorf Galaxy 170R CO_2 incubator. The camera was cooled to -95°C to minimize dark noise as much as possible. Images were formed from integrated photon counts over 25 minutes every 30 minutes using Metamorph Software (Molecular Devices). Exported images were composed into image stacks and regions of interest (each well of the plate) were quantified in a time series using a custom script in NIH ImageJ software, using the "Multi-measure" plugin. Bioluminescence data traces were analysed using a modified version of the R script "CellulaRhythm" (Hirota et al., 2008), which allows baseline subtraction and detrending using polynomial fitting to the signal.

NAD(P)/NAD(P)H assays

NAD^+/NADH and $NADP^+$/NADPH were measured using the NAD/NADH-Glo™ Assay and NADP/NADPH-Glo™ Assay kits respectively (Promega). U2OS cells were grown to confluence in 96-well plates and, 24 h before measurement, spent medium was changed to fresh medium and the concentration of drugs indicated in the main text were added to the cells. Just before NAD(P)/NAD(P)H assays, cell medium was removed and replaced by 50 µl of PBS solution. The NAD^+/NADH and $NADP^+$/NADPH ratios were then measured from the same set of cells following manufacturer's instructions. For the NADPH time course, Bmal1:luc U2OS cells were grown to confluence, synchronized with 100 nM dexamethasone for 15 min (Balsalobre et al., 2000). At the indicated time points, cells were collected, flash-frozen and stored at -80°C until biochemical analysis.

Gel electrophoresis and immunoblotting

For time course experiments, *Bmal1*:*luc* U2OS cells treated with 5mM 6AN or control (DMSO) were synchronized with a dexamethasone shock and lysed in 1x SDS sample buffer at the indicated time points. Lysates were incubated at 95 °C for exactly 10 min, flash-frozen and transferred to –80 °C until immunoblot analysis. NuPAGE Novex 10% Bis-Tris gradient gels were run according to the manufacturer's protocol with a nonreducing MES SDS buffer system. Protein transfer to nitrocellulose for blotting was performed and the membrane was then washed briefly and then blocked for 60 min in 0.5% wt/wt BSA/nonfat dried milk (Marvel) in Tris-buffered saline/0.05% Tween-20 (TBST). After three brief washes in TBST, membranes were incubated in anti-PRDX-SO3 (LF-PA0004, Thermo Fisher Scientific) or anti-ACTB (sc-47778, Santa Cruz Biotechnology) diluted in blocking buffer (0.5% milk/BSA) overnight at 4 °C. The following day, membranes were washed for 5 min three times (in TBST) and then incubated with 1:10,000 HRP-conjugated secondary antibody (Sigma-Aldrich) for 60 min. Three more 5-min washes were then performed before performing chemiluminescence detection. Densitometric quantification of images was performed using NIH ImageJ software. Immunblot signals were first normalized with loading control (actin) and then normalized to the average for each replicate.

Metabolic rate measurements

Extracellular acidification rate (ECAR), a surrogate for glycolysis rate, and oxygen consumption rate (OCR), an indication of mitochondrial activity, were measured in a Seahorse XF24 Bioanalyser using the manufacturer's

instructions. Briefly, U2OS cells were grown to confluence in Seahorse Biosciences 24-well culture plates and, 1 h before measurement, growth medium was replaced by XF Assay Medium (Seahorse Biosciences) supplemented with 25 mM glucose and 1 mM pyruvate, containing 5mM 6AN or control (DMSO).

Metabolomics

Around 100 Metabolites from *Bmal1*:*luc* U2OS samples were separated using Acquity ultra pressure liquid chromatography (UPLC) and analyzed using XEVO-TQ-S Triple Quadrupole mass spectrometer (Waters, USA). Approximately 1 million cells incubated with 0.5% DMSO, 5mM 6AN or 50 μM of DHEA for 24 hours (N=3 replicates per group) were washed with PBS and deionized water very briefly, and subsequently quenched in liquid nitrogen. Metabolites were extracted by adding 20 μL of labeled internal standard mix and 1ml of cold extraction solvent (80/20 acetonitrile/H2O:1% formic acid). Extracts were vacuum filtered (pressure 300–400 mbar for 2.5 min; Hamilton micro lab robot) and injected 5 μL into the LC system. A detailed description of instrument parameters is given elsewhere (Roman-Garcia et al., 2014).

RNA isolation and quantitative PCR

For mRNA expression time course, *Bmal1*:*luc* U2OS cells were synchronized with dexamethasone (Figure S4A) and cultured in DMEM as described above, supplemented with 5 mM 6AN or a matched amount of DMSO (0.5%) as a control. At the time points indicated in the main text, RNA was extracted with TRI-Reagent® in triplicate and purified with Direct-zol™ RNA MiniPrep kit (Zymo Research). The RNA samples were reverse-transcribed into cDNA using the High Capacity cDNA Reverse Transcription Kit (Life Technologies), following the manufacturer's instructions, using 0.3-1μg total RNA per reaction. The resulting cDNAs were used in duplicate 7 μl PCR reactions, set up as follows: 3.5 μl TaqMan Gene Expression Master Mix (Life Technologies), 0.35 μl validated Taqman Gene Expression Assay (Life Technologies) (see Table S2 for complete list), 1.15 μl nuclease-free water and 2 μl cDNA. Real-time PCR was performed with an ABI 7900HT (Applied Biosystems) system. The relative levels of each mRNA were calculated by the $2^{-\Delta\Delta Ct}$ method and normalized to the corresponding *ACTB* levels. For straightforward comparison, relative expression for each gene was then scaled across the treatment and control conditions.

RNA-seq library preparation

Ribosomal RNA was removed from total RNA using selective depletion of abundant RNA (Adiconis et al., 2013; Morlan et al., 2012). RNA was then purified using Serapure beads and fragmented by incubation for 15 minutes at 94°C in 1X NEBNext First Strand Synthesis Reaction Buffer and cDNA was made according to suppliers instructions (NEB #B6117S). Second strand synthesis was performed by incubating the cDNA reaction with 200 μM dNTP mix, 1.3U E. coli DNA Ligase (NEB# M0209L), 0.5U E. coli DNA Polymerase I (NEB# M0205L), 1U RNAse H (NEB# M0297S). For library preparation, Illumina TrueSeq platform compatible dual-indexed multiplex library protocol was followed. Briefly, the end repair was performed by incubating at 20°C for 30 minutes with 0.7U T4 Polynucleotide Kinase (NEB# M0201L), 2.3U T4 DNA Polymerase (NEB# M0203L), 0.2U DNA Polymerase I, Large (NEB# Klenow) Fragment (NEB# M0210L) followed by a clean-up with 1.8X AMPure XP Magnetic Beads. dA-tailing of end-repaired fragments was then performed by incubation for 30 minutes at 37°C in 1X NEB Buffer 2, 0.2mM dATP, 1.25μl Klenow 3'->5' exo- (NEB# M0212L) in a 50μl reaction. Immediately, adaptor ligation was performed by incubation for 20 minutes at 20°C in 1X Quick ligase buffer, 50mM EDTA, 0.6μM TruSeq dual index adapter, 1.5μl Quick ligase (NEB# M2200L) in a 50μl reaction. Adapter-ligated DNA was subsequently cleaned-up by 1X AMPure XP Magnetic Beads twice. Finally, libraries were amplified using the following reaction: 20μl adapter-ligated DNA, 5μl Truseq Primer Cocktail (5μM) and 25μl 2X Kapa Hifi Hotstart Ready Mix. Reactions were amplified with 15 cycles of amplifications and were cleaned-up using AMPure XP Magnetic Beads.

RNA-seq analysis

Sequencing reads were filtered for rRNA sequences by removing all reads mapping to the 45S precursor rRNA sequence using Bowtie v1.1.1 (Langmead et al., 2009). Filtered reads were aligned to the human reference genome (hg38) using TopHat v2.1.0 (Kim et al., 2013). Reads were assembled into transcripts using UCSC known genes as reference and their abundance estimated using Cufflinks/Cuffmerge/Cuffquant/Cuffnorm v2.2.1 (Trapnell et al., 2010). For estimation of circadian parameters, we restricted our analysis to transcripts with an abundance greater than 0.05 FPKM. Temporal profiles were linearly detrended and the RAIN algorithm was used to detect rhythmic transcripts using the following parameters: minimal period=20, maximal period=28, method=longitudinal and p-value=0.01 (Thaben and Westermark, 2014). As validation, two alternative algorithms, Fisher test (Rey et al., 2011) and ARSER (Yang and Su, 2010) (minimal period=20, maximal period=28, default period=24), were used to detect circadian transcripts. To estimate the phase and period of transcripts, we performed nonlinear least square regression of cosine waves, similarly to the R script "CellulaRhythm" (Hirota et al., 2008). GO analyses were performed using the Gene List Analysis Tool from the

PANTHER database (Mi et al., 2016). The list of NRF2 target genes was compiled by assembling the list of genes assigned to the 849 NRF2 ChIP-Seq peaks from (Chorley et al., 2012) and the human homologs of NRF2 basal and inducible genes identified in (Malhotra et al., 2010).

Chromatin Immunoprecipitation

Chromatin Immunoprecipitation (ChIP) was performed on *Bmal1*:*luc* U2OS using a modified version of an established protocol (Mortazavi et al., 2006). *Bmal1*:*luc* U2OS cells were grown to confluence in 150 mm dishes and one dish was used per immunoprecipitation. 24 h after treatment with 5 mM 6AN or control, cells were cross-linked by adding formaldehyde to a final concentration of 1% for 5 min. Cross-linking was stopped by adding glycine to a final concentration of 0.125 M. Then, cells were washed once with PBS, scraped off the dish and re-suspended in lysis buffer (5 mM 1,4-piperazine-bis-[ethanesulphonic acid] pH 8.0, 85 mM KCl, 0.5% NP-40, Complete Protease Inhibitor Cocktail (Roche)) and centrifuged to collect the crude nuclear preparation. Nuclei were washed once with lysis buffer and then re-suspended in RIPA buffer (1X PBS, 1% NP-40, 0.5% sodium deoxycholate, 0.1% sodium dodecyl sulphate (SDS), Complete Protease Inhibitor Cocktail (Roche)). Cells were then sonicated in a Bioruptor (Diagenode) on the HIGH setting for a total time of 60 minutes (30 seconds ON, 30 seconds OFF). Samples were centrifuged at 14,000 rpm in a bench-top microfuge for 15 min at 4°C and the supernatant containing the sonicated chromatin was collected.

For immunoprecipitation, primary antibodies were coupled to Protein G magnetic beads (Dynal/Invitrogen) for 4 h at 4°C on a rotating wheel. The following validated ChIP-grade antibodies were used: anti-CLOCK (NB100-126; Novus Biologicals), anti-BMAL1 (ab3350; Abcam), anti-H3K9ac (ab4441; Abcam), anti-H3K4me3 (#39159; Active Motif), anti-p300 ((C-20) sc-585 Santa Cruz Biotechnology and anti-NFE2L2 Antibody EP1808Y OriGene Technologies). Antibody-coupled beads were then added to the sonicated chromatin preparation and incubated at 4°C overnight on a rotating wheel. A small aliquot of the chromatin preparation was kept as "input" chromatin. The magnetic beads were washed five times with wash buffer (100 mM Tris, 500 mM LiCl, 1% NP-40, 1% sodium deoxycholate) and washed once with TE (10 mM Tris at pH 8.0, 1 mM EDTA). After washing, the bound DNA was eluted by heating the beads to 65°C in elution buffer (100 mM NaHCO3 and 1% SDS) for 1 h. The eluted DNA and input DNA were incubated at 65°C overnight to reverse formaldehyde cross-links. QIAquick PCR Purification Kit (Qiagen) was used to purify DNA from the chromatin preparation. Quantitative real-time PCR using the SYBR Green detection system was performed with an ABI 7900HT (Applied Biosystems) system. The amount of immunoprecipitated DNA at each genomic locus was normalized to the input DNA and expressed as percentage input. Primers used are listed in Table S2.

ChIP-Seq analysis

DNA reads were mapped to the human genome (hg38 from UCSC database) using Bowtie2 v2.2.1 (Langmead and Salzberg, 2012). If several reads mapped at the same genomic position, we considered this as a PCR duplicate and only one read was kept for the rest of the analysis. To normalize for differences in sequencing depth among the time points, the number of tags was rescaled by the total number of mapped tags. HOMER software suite was used to detect, quantify and annotate ChIP-Seq peaks (Heinz et al., 2010). HOMER was also used to analyze enriched motifs in ChIP-Seq peaks.

Organotypic slice culture and bioluminescence

All animal experimentation was licensed by the UK Home Office under the Animals (Scientific Procedures) Act 1986, and according to the European Parliament and Council of the European Union Directive 2010/63/EU. Local Ethical Review was also conducted by the University of Cambridge. Prior to use in experiments, animals were group-housed in individually-ventilated cages under a 12:12 light:dark cycle with food and water available *ad libitum*. Ambient temperature was monitored daily, and maintained at 22±2°C. SCN slices were extracted from 8-12 week old adult *mPer2*^{Luc} mice (Yoo et al., 2004). Mice were humanely sacrificed by cervical dislocation, and the brain immersed in cold dissection medium (Gey's Balanced Salt Solution [Sigma G9779], 5.0 g/l glucose, 100 nM MK-801 [Sigma M107], 50 μM DL-AP5 [Tocris 0105], and 3 mM $MgCl_2$ [Fluka 63020]). Liver slices were immersed in cold Hank's Balanced Salt Solution without Ca^{2+} and Mg^{2+} (Life Technologies 14170-122), and sectioned at a thickness of 500 μm on a tissue chopper (McIlwain, Mickle Laboratory Engineering). SCN slices were cut in the coronal plane at a thickness of 200 μm on a vibrating microtome (Campden Instruments, 7000smz-2). Slices were cultured on a membrane (Merck Millipore, PICM0RG50) in a sealed dish containing "modified Air Medium" (DMEM (Sigma) with 30 mM glucose, 20 mM HEPES, 0.035% NaHCO3, 25% (v/v) Heat-inactivated Horse Serum (Life Technologies 26050-088), 1X GlutaMAX™ (Life Technologies 35050-038), 1X B-27® Supplement (Life Technologies 17504-044), 1 mM Luciferin (Biosynth L8220), and 1X MycoZap™ Plus-PR (Lonza)) with either 500 μM 6AN or 0.5% DMSO (control). Slices were then transferred to custom imaging incubators for whole explant bioluminescence recording, or microscopes for single-cell bioluminescence imaging.

Whole explant imaging of SCN and liver slices was performed using an Andor iKon-M 934 cooled CCD camera mounted CO_2 incubator at 37°C (see "Cell culture and bioluminescence assays section" above for further details). Single-cell images were recorded from SCN slices placed into an Okolab stage-top heated chamber (37°C) mounted on an inverted Nikon Eclipse Ti-E microscope equipped with an electron- multiplied CCD (EM-CCD) camera (Hamamatsu ImagEM 1K, C9100-14). Images were captured on NIS Elements AR v4 software (Nikon). Luminescence recordings proceeded for 4-5 days, after which the culture medium was exchanged with "modified Air Medium" (without 6AN or DMSO), and recording resumed for another 5 days. All images were exported into ImageJ software (National Institutes of Health) for analysis. Bioluminescence data traces were analysed using a modified version of the R script "CellulaRhythm" (Hirota et al., 2008). Statistical analysis was performed in GraphPad Prism 5 (GraphPad Software).

Single-cell bioluminescence assays

For single-cell bioluminescence recordings, *Per2*:*luc* U2OS cells were plated to confluency on 35mm glass-bottom dishes (FD35-100, WPI Inc., Sarasota, FL) and synchronised by changing medium to "Air Medium." Dishes were sealed and placed into a heated Okolab chamber (37°C) mounted on an inverted Nikon Eclipse Ti microscope equipped with an EM-CCD camera (Hamamatsu ImagEM-1K). Images were captured on NIS Elements (Nikon) software continuously for 5 days. Images were formed from integrated photon counts over 22 minutes every hour. Exported images were composed into image stacks and regions of interest around individual cells were quantified as described above.

Fly behavioural assays

Wild type Canton-S flies were bred and grown on standard yeast cornmeal agar medium at 25°C in 12h:12h light:dark cycles (LD). For behavioural recording experiments, individual flies were placed into wells of a 96-well plate following brief exposure to CO_2 anaesthesia. Each well contained an equal volume of assay medium (5% Sucrose, 1% Agar), supplemented with 6AN or control (DMSO) at concentrations indicated in the main text. Since the 6AN was dissolved in DMSO (1M stock solution), DMSO proportionally increased as 6AN dose increased, and therefore control flies were dosed with equivalent (matched) concentrations of DMSO to account for this. Although the concentrations of drug were high in comparison to those used in our cell and tissues studies, it is important to note that the Drosophila were ingesting agar dosed with the drug and therefore received a much lower effective concentration. Using a custom made infra-red video recording system, the locomotor activity of individual 4-7 day old flies was recorded in constant darkness (DD) following 2 days of entrainment in LD cycles (which were not recorded). The videos were processed using Ethovision XT v10 software (Noldus) to quantify the locomotor activity of the flies. We measured the distance travelled per minute as a measure of the flies' locomotor activity (with a cut off of 0.2 mm of movement in any direction as a significant movement). The activity data of our custom-made system closely matches that produced by the beam crossing method used in the Drosophila Activity Monitoring System (TriKinetics). Individual or experimental median activity records, periodic activity profiles, and autocorrelation were generated using custom R scripts.

SUPPLEMENTAL REFERENCES

Adiconis, X., Borges-Rivera, D., Satija, R., DeLuca, D.S., Busby, M.A., Berlin, A.M., Sivachenko, A., Thompson, D.A., Wysoker, A., Fennell, T., et al. (2013). Comparative analysis of RNA sequencing methods for degraded or low-input samples. Nat Methods *10*, 623–629.

Balsalobre, A., Marcacci, L., and Schibler, U. (2000). Multiple signaling pathways elicit circadian gene expression in cultured Rat-1 fibroblasts. Curr Biol *10*, 1291–1294.

Hastings, M.H., Reddy, A.B., McMahon, D.G., and Maywood, E.S. (2005). Analysis of circadian mechanisms in the suprachiasmatic nucleus by transgenesis and biolistic transfection. Methods Enzymol *393*, 579–592.

Heinz, S., Benner, C., Spann, N., Bertolino, E., Lin, Y.C., Laslo, P., Cheng, J.X., Murre, C., Singh, H., and Glass, C.K. (2010). Simple combinations of lineage-determining transcription factors prime cis-regulatory elements required for macrophage and B cell identities. Mol Cell *38*, 576–589.

Kim, D., Pertea, G., Trapnell, C., Pimentel, H., Kelley, R., and Salzberg, S.L. (2013). TopHat2: accurate alignment of transcriptomes in the presence of insertions, deletions and gene fusions. Genome Biol *14*, R36.

Langmead, B., and Salzberg, S.L. (2012). Fast gapped-read alignment with Bowtie 2. Nat Methods *9*, 357–359.

Langmead, B., Trapnell, C., Pop, M., and Salzberg, S.L. (2009). Ultrafast and memory-efficient alignment of short DNA sequences to the human genome. Genome Biol *10*, R25.

Mi, H., Poudel, S., Muruganujan, A., Casagrande, J.T., and Thomas, P.D. (2016). PANTHER version 10: expanded protein families and functions, and analysis tools. Nucleic Acids Res *44*, D336–D342.

Morlan, J.D., Qu, K., and Sinicropi, D.V. (2012). Selective depletion of rRNA enables whole transcriptome profiling of archival fixed tissue. PLoS ONE *7*, e42882.

Roman-Garcia, P., Quiros-Gonzalez, I., Mottram, L., Lieben, L., Sharan, K., Wangwiwatsin, A., Tubio, J., Lewis, K., Wilkinson, D., Santhanam, B., et al. (2014). Vitamin B_{12}-dependent taurine synthesis regulates growth and bone mass. J. Clin. Invest. *124*, 2988–3002.

Trapnell, C., Williams, B.A., Pertea, G., Mortazavi, A., Kwan, G., van Baren, M.J., Salzberg, S.L., Wold, B.J., and Pachter, L. (2010). Transcript assembly and quantification by RNA-Seq reveals unannotated transcripts and isoform switching during cell differentiation. Nat Biotechnol *28*, 511–515.
